# Directional syncretism without directional rules

**DOI:** 10.1007/s11525-025-09443-4

**Published:** 2025-06-06

**Authors:** Johannes Hein, Andrew Murphy

**Affiliations:** 1 Institut für deutsche Sprache und Linguistik, Humboldt-Universität zu Berlin, Unter den Linden 6, 10177 Berlin, Germany; 2 USC Department of Linguistics, University of Southern California – Los Angeles, 3601 Watt Way, Grace Ford Salvatori Hall 301, Los Angeles, CA 90089-1693 USA

**Keywords:** Syncretism, Directional syncretism, Distributed Morphology, Impoverishment, Bidirectional syncretism, Markedness

## Abstract

Certain patterns of directional syncretism, in particular bidirectional syncretism, have been argued to necessitate the power of directional rules that create a form dependency between two cells in a paradigm. The existence of such patterns, divergent bidirectional syncretism in particular, has been claimed to be fatal for the approach to syncretism adopted in Distributed Morphology that holds that (directional) syncretism always involves a retreat to less specific and therefore less marked exponents. In this paper, we will demonstrate that this is not the case. Apparently challenging cases of directional syncretism can be adequately handled on the view that impoverishment may have two outcomes: deletion of features or insertion of contextually unmarked values. Once this view is adopted, bidirectionality is no longer a problem. Furthermore, we argue that this view allows us to still maintain the claim that syncretism is universally markedness decreasing if markedness is defined over insertion contexts rather than exponents.

## Introduction

A key goal of many theoretical approaches to inflectional morphology is to account for the lack of one-to-one correspondence between syntactic contexts and phonological forms, otherwise known as *syncretism*. Assuming that the task of such a theory is to provide the set of rules or constraints governing which morphological form is associated with a given combination of morphosyntactic features, syncretism poses the challenge of how to account for this mismatch. It is here that so-called realizational theories of morphology, i.e. those which take inflection to consist in the expression of an already given morphosyntactic feature set, differ in the way they analyze certain syncretic patterns. One of the major dividing lines between approaches such as Paradigm Function Morphology (Stump, 1993, 2001, 2016; Bonami & Stump, 2017) and Network Morphology (Corbett & Fraser, 1993; Brown & Hippisley, 2012), on the one hand, and Distributed Morphology (Halle & Marantz, 1993, 1994; Halle, 1997; Harley & Noyer, 2003; Embick & Noyer, 2007) and Nanosyntax (Starke, 2009; Caha, 2009, 2018, 2020; Baunaz et al., 2018) on the other is whether or not they view the paradigm as a primitive of the theory or an epiphenomenon to be derived by other principles of the grammar.

It is in this regard that a certain sub-type of syncretism is particularly relevant, namely *directional syncretism*. In directional syncretism, it is not just that two or more morphosyntactic contexts show no distinction in form, but it seems that one context has ‘borrowed’ the form of another (Carstairs, 1987). The question of how to derive this phenomenon has already been the subject of some debate in the literature (Zwicky, 1985; Stump, 1993, 2001; Bobaljik, 2002; Baerman, 2004; Wunderlich, 2004). In theories that recognize the paradigm as a component of the grammar, it is possible to derive the effect of borrowed forms by means of a ‘directional rule’ which states that two values share the form of one of the component members (Baerman et al., 2005: 134). The most common instantiation of a directional rule is a so-called ‘rule of referral’ that acts as a pointer to another cell in the paradigm, i.e. an instruction to use the form associated with that cell for the realization of a different cell. Such a rule straightforwardly captures the phenomenon of ‘borrowed’ forms in directional syncretism.

In syntacticocentric theories eschewing the paradigm or comparable unrestricted form-meaning mappings, syncretism is the result of manipulating feature bundles or syntactic structures. This is the case in Distributed Morphology, where syncretism is most commonly treated as the result of underspecification (a rule of exponence may fail to mention certain features) and/or impoverishment, whereby the features are removed from the set of features to be realized prior to insertion of forms. In this approach directional effects cannot be expressed as such, but must instead be the result of an impoverished feature set. Since impoverishment typically leads to insertion of a less-specific matching exponent, the prediction of a DM account is that syncretism will involve a so-called ‘retreat to the general case’ or insertion of a less-specific exponent in lieu of the expected one. As noted by Bobaljik (2002: 64), this has the consequence that syncretisms in DM will always be ‘neutralizations toward lesser marked forms’.

It is with regard to how best to model directional effects in syncretism that patterns of ‘bidirectional syncretism’ become particularly relevant. Bidirectional syncretism (BDS) involves a paradigm that contains two distinct instances of directional spreading. An example of one such kind of bidirectional syncretism, identified by Baerman (2004) as ‘divergent bidirectional syncretism, is shown below in (1) for the so-called ‘second declension’ in Latin. Throughout, we represent the target cell of a directional syncretism in ).

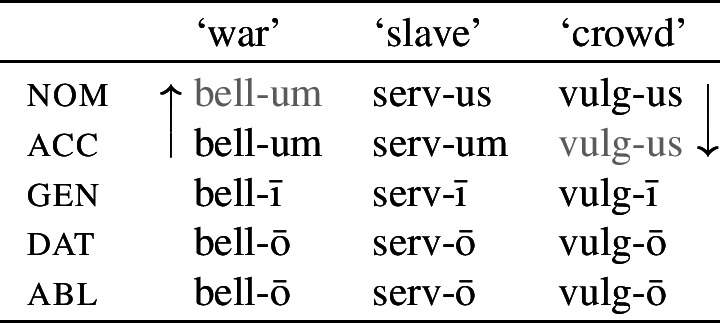

In this paradigm, we have what looks like directional spreading of the accusative suffix *-um* to the nominative with nouns like ‘war’, while the spreading of *-us* goes in the opposite direction (from nominative to accusative) with other nouns such as ‘crowd’.

This particular bidirectional pattern has been argued to be problematic for impoverishment-based theories in two respects. First, Baerman (2004) notes that, for theories employing underspecification, bidirectional patterns require specifying overlapping domains for the two exponents. In the case of divergent BDS, the marker specifications must be fully overlapping and this presents a problem that can only be solved by appealing to a directional rule. Second, it has been argued that bidirectional syncretism is a challenge to the view that directional syncretism necessarily involves a reduction in markedness (Stump, 2001). For this reason, bidirectional syncretism (particularly the divergent type) has been taken as one of the ‘counterexamples to the DM doctrine on syncretism’ (Spencer, 2019: 25).

To the best of our knowledge, there has so far been no attempt from the side of those working in Distributed Morphology to respond to the challenge posed by bidirectional syncretism, particularly the divergent type.^1^ In this paper, we will take up the challenge of analyzing bidirectional syncretism and show that, contrary to such claims, the relevant patterns can be derived in a way that is in line with DM’s central assumptions about syncretism. Following Noyer’s (1998) proposal that impoverishment may have two outcomes (either deletion or a markedness-reducing feature change), even the problematic divergent BDS can be readily analyzed. Furthermore, we will demonstrate that the assumption that syncretism involves a relative decrease in markedness, what we call the *Unmarkedness Hypothesis*, can be upheld in the light of apparent counterevidence if we acknowledge three levels of markedness and state the generalization over feature sets rather than exponents.

What is more, we will argue that the resulting theory can remove indeterminacy in the analysis of bidirectional syncretism, specifically because markedness will constrain the choice of which of the two syncretisms must be treated as a genuine directional syncretism rather than a neutralization to a less specific form. In addition, we will address some further challenging patterns of syncretism in Dhaasanac (Baerman, 2004; Baerman et al., 2005) and Kashmiri (Stump, 2016) that have been argued to require recourse to morphomic devices (Trommer, 2016) and show that, once we have Noyer’s enriched view of impoverishment, these patterns can also receive a natural analysis in terms of (bi-)directional syncretism.

The paper is structured as follows. In Sect. 2, we review the different subtypes of syncretism identified in Stump (2001), including directional syncretism, and recap some of the theoretical discussion about the proper analysis of directional syncretism, in particular Bobaljik’s (2002) reply to claims in (Stump, 1993, 2001). In Sect. 3, we focus on bidirectional syncretism in detail, discussing both the convergent and divergent types and how they could be analyzed in Distributed Morphology. Here, we will concur with Baerman (2004) that the divergent type does indeed pose a serious challenge for an approach without directional rules. We go on to argue, however, in Sect. 4, that this challenge can be overcome by adopting the empirically well-supported view developed in Noyer (1998). We first lay out the empirical motivation that Noyer provides for this approach, coming from interacting patterns of directional syncretism in Nimboran. We then go on to show how this machinery can readily derive the problematic divergent bidirectional pattern. Section 4.6 discusses two further implications of the analysis. First, we illustrate that Noyer’s analysis allows for a reanalysis of convergent BDS as unidirectional syncretism. Additionally, we show how previously recalcitrant patterns of diagonal syncretism in Dhaasanac and Kashmiri can be understood in terms of ‘hidden’ bidirectionality. Finally, Sect. 5 concludes.

## Directional syncretism

Syncretism refers to a one-to-many relation between morphological forms and morphosyntactic contexts. Stump (2001) proposed a distinction between at least three types of syncretism, namely *unstipulated syncretism*, *symmetrical syncretism* and *directional syncretism* (which can be further sub-classified, as we discuss below). To see this, consider the paradigm in (2) showing the plural adjectival declension of the adjective *słaby* (‘weak’) in Polish (Joanna Zaleska, p.c.).

(2)
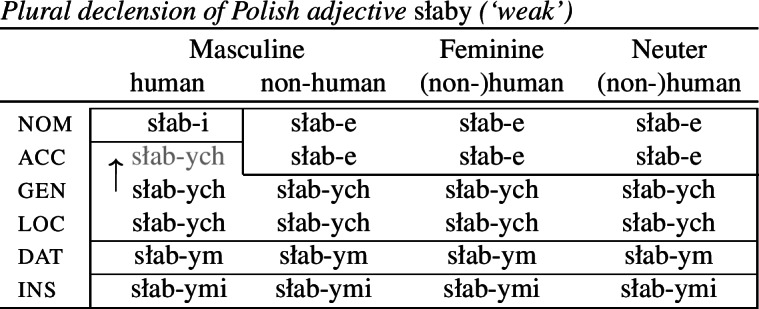

The suffixes *-ym* and *-ymi* are instances of what Stump calls *unstipulated syncretism* because they each realize a single case value, but are completely underspecified for gender. Other syncretisms cover contexts corresponding to multiple feature values. The suffix *-ych* spans both the genitive and locative rows in the paradigm. This means that the apparently unrelated feature values gen and loc must be treated as a homogeneous class for the purposes of morphological realization. The same is true for *-e*, which is syncretic for the values nom and acc. Stump refers to this as *symmetrical syncretism* since neither of the feature values involved can be seen as the more basic value on which the other is dependent. This differs from the final type of syncretism, *directional syncretism*, where it is possible to identify an asymmetry between the syncretic cells such that the form used in one context appears to be borrowed from another context. For example, the masculine human accusative has the form *-ych* that is otherwise associated with the stipulated class of gen and loc. We can therefore say that the genitive/locative form ‘spreads’ to the accusative in this context, or that there is a borrowing or ‘take-over’ (in the sense of Carstairs, 1987) of the genitive/locative form in the accusative.

### Two approaches to syncretism

As mentioned in the introduction, there is a fundamental difference in the status of the paradigm in some (inferential-)realizational approaches such as Paradigm Function Morphology, Network Morphology and A-Morphous Morphology in contrast to syntacticocentric realizational models like Distributed Morphology. While the former typically treat the paradigm as a primitive of the theory, this view is rejected in DM and Nanosyntax. This difference becomes particularly relevant when we consider how the two kinds of approaches handle directional syncretism.

To appreciate this difference, let us consider how each kind of syncretism in the Polish data in (2) could be analyzed. A straightforward realizational analysis in a theory acknowledging paradigms could look as follows (see e.g. Baerman, 2004; Baerman et al., 2005). Unstipulated syncretism is straightforward, as realization rules only need to refer to a single case value for a given form (3).

(3)
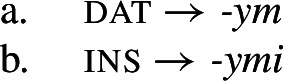

For symmetrical syncretism, Baerman (2004) suggests defining an abstract ‘index’ X (4a) or Y (4c) that corresponds to the unification of two values. Realization rules may then refer to this stipulated class via the index (4b,d).

(4)
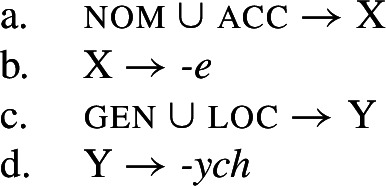

As for directional syncretism, a paradigm-based approach implements the idea of a ‘take-over’ directly by means of a *rule of referral* (Zwicky, 1985; Stump, 1993, 2001). Rules of referral can be viewed as pointers from one cell in a paradigm to another. For example, the spreading of the genitive/locative form to the accusative in the human masculine cell in (2) can be captured by the following rule of referral:

(5) *Rule of referral (Polish)*
  The accusative human masculine cell takes the same form as the genitive human masculine (acc hum masc ⇒ gen (hum masc)).

This rule will have to count as highly specific in order to override the expected realization rule for this cell in (4b).

In DM, where syncretism is derived by the postsyntactic manipulation of feature bundles created in the syntax (see Kramer, to appear for an overview), analyzing the paradigm in (2) requires somewhat different assumptions about feature structure. In order to derive symmetrical syncretism, it is commonplace in DM to decompose traditional grammatical categories such as case into component sub-features (Jakobson, 1962; Bierwisch, 1967). These features are sometimes assumed to be functionally-motivated (e.g. [±governed], [±oblique]), but for purely illustrative purposes, we adopt arbitrary features such as [±a] and [±b]. Let us then adopt the decomposition of case features in (6).

(6)
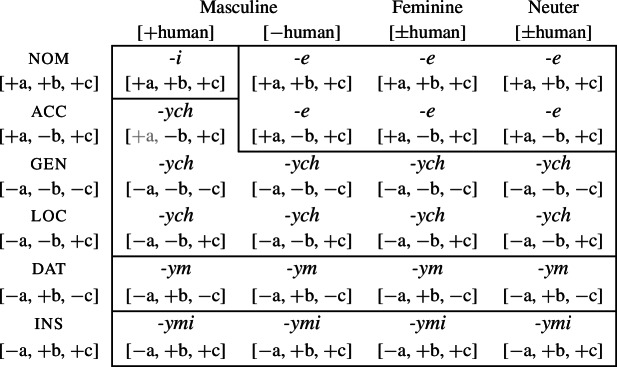

In this analysis, paradigm cells actually correspond to feature bundles (or complex heads) derived by the syntax. As such, the paradigm itself is epiphenomenal. Nevertheless, for expository purposes we will arrange all possible combinations of case features in a paradigm such as (6).^2^ For each of these feature combinations, one must determine what the relevant realization rule, or Vocabulary Item, is. The highly specific marker *-i* occurs in a single context and is thus specified for a full set of case, animacy and gender features (7a). For unstipulated syncretism, i.e. the markers *-ym* and *-ymi*, the Vocabulary Items will only realize the relevant case features (7b,c). They are underspecified for animacy and gender features, allowing them to appear in any of the columns in (6). Symmetrical syncretism involves underspecification for a sub-feature of a traditional grammatical category such as case. For example, *-e* appears in both nominative and accusative contexts, so we can assume that it is only specified for the sub-features that uniquely identify those two cases to the exclusion of all others, that is, [+a, +c] (7d). This means that *-e* will be inserted in the six contexts in (6). However, this specification also means that *-e* is eligible for insertion in masculine human nominative and accusative contexts, respectively. For the masculine human nominative, both *-i* (7a) and *-e* (7d) are possible candidates for insertion given the Subset Principle (Halle & Marantz, 1993; Halle, 1997). Since *-i* realizes more features of the insertion context than *-e*, it will take precedence over *-e* given the standard Specificity/Elsewhere Principle.

(7)
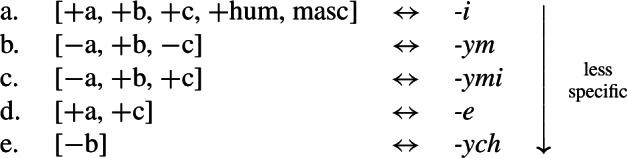

This explanation does not account for why *-e* is not inserted in the masculine human accusative, however. Given the distribution of *-ych*, it must be highly underspecified so as to be eligible for both genitive and locative in addition to accusative contexts. This means that it can only realize the sub-feature value they have in common, namely [−b] (7e).

This alone cannot account for why *-ych* appears to block *-e*, however, since the latter is more specific. As mentioned above, the spreading of *-ych* to the accusative is an instance of directional syncretism. In DM, directional syncretism is analyzed as bleeding of insertion of an expected marker by manipulation of the insertion context. Most often, this is achieved through deletion of features via an impoverishment rule. In order to effect spreading of *-ych* to a context in which we would otherwise expect to find *-e*, we can remove one of the features realized by *-ych* from the insertion context, namely [+a], by means of the following impoverishment rule:^3^

(8)
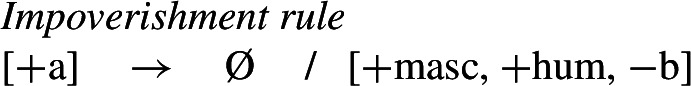

As a result, *-e* is no longer eligible for insertion into the modified context [−b, +c], given the Subset Principle. Impoverishment therefore bleeds insertion of the originally expected, most specific, exponent (*-e*) in favour of a matching, albeit less specific exponent (*-ych*, in this case). As such, directional syncretism (and indeed syncretism in general) is assumed to involve insertion of a less specific matching exponent over a more specific one, or what has been called a *Retreat to the General Case* (Halle & Marantz, 1993, 1994).

### Rules of referral vs. impoverishment

There has already been some discussion of the relative virtues of these two approaches to syncretism in the literature. The main advantage of rules of referral is that they are powerful enough to describe any pattern of syncretism and can therefore handle the full range of cross-linguistic variation (see e.g. Stump, 2001; Baerman, 2004; Baerman et al., 2005). The DM alternative employing impoverishment, on the other hand, has been argued to have the virtue of being more restrictive in only permitting some but not all kinds of directional syncretism (Bobaljik, 2002; Kramer, 2016). If both theories can be shown to achieve the same empirical coverage, then the more restrictive theory should be preferred (Noyer, 1998). The important question becomes whether there are empirical arguments which can help to distinguish between the two theories.

Stump (1993) argued for the necessity of rules of referral on the basis of the Macedonian data in (9) (also see Stump, 2001 for a similar argument based on Bulgarian). The Macedonian verbs in this paradigm have three inflectional suffixes, each identified as a distinct position class. Position I is a theme vowel, position II is a tense/aspect suffix and position III hosts person/number agreement morphology. In both the imperfect and aorist, which Stump treats as a natural class (e.g. [+past]), the 2sg and 3sg forms are identical, e.g. *padneše* in the 2sg/3sg imperfect. Stump assumes that *-še* realizes 3rd person singular imperfect and *-v* is a non-3rd person past tense (imperfect/aorist) marker. Given this, we would expect both to find *-v* and not to find *-še* in the 2nd singular imperfect. Instead, we have the form *padneše*, suggesting that there has been a simultaneous take-over of 3rd singular *-Ø* in position II and of 3rd singular *-še* in position III. We also see the same effect in the aorist where the 2sg takes the form of the 3sg. (Note that we only mark zero exponents in the relevant cases of directional syncretism.)

(9)
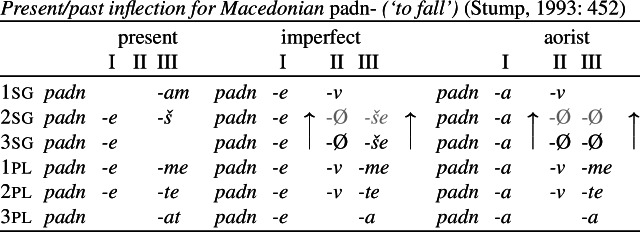

Stump accounts for this apparent conspiracy between position classes by appealing to whole-word syncretism. His rule of referral in (10) stipulates that the entire word form of the 2nd singular must be identical to the 3rd singular past, thereby making the directional syncretism across position classes non-accidental.

(10) *Rule of referral (Macedonian)*
  In the past tenses, the second person singular has the same form as the third person singular (2sg past ⇒ 3sg past).

According to Stump, the relatedness of the two take-overs would be a ‘mere coincidence’ on an alternative underspecification approach (Stump, 1993: 453). He therefore argues that the ability to account for the apparent conspiracy of multiple directional syncretisms in deriving a whole-word syncretism is an advantage of using rules of referral.

Bobaljik (2002) responds to this by pointing out that an impoverishment-based analysis in Distributed Morphology also avoids the conspiracy problem of a pure underspecification approach. Let us assume that the 2nd singular imperfect form *padneše* derives from the syntactic structure in (11), where each functional head corresponds to a position class in (9).

(11)

The various functional heads in this structure may then be realized by Vocabulary Items. The relevant VIs for the Agr and Tense nodes are given in (12) and (13).

We assume that the theme vowel is a realization of *v*, but will ignore this for present purposes.

(12)
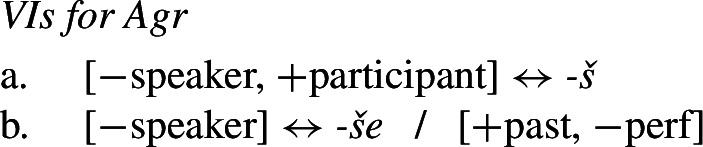

(13)
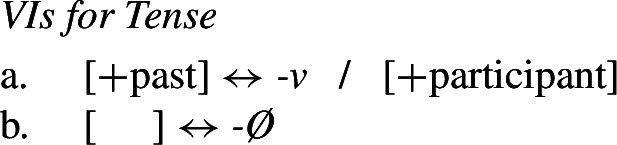

Recall that the desired surface form for the 2nd singular imperfect is *padn-e-Ø-še*. Given the rules in (12) and (13), however, we would expect Agr to be realized as *-š* by (12a) and Tense to be realized by *-v* (13a). This means that we incorrectly predict the relevant form to be *padn-e-v-š. In order to effect directional syncretism in position III, we have to bleed insertion of the expected marker in (12a). This can be achieved by the impoverishment rule in (14), which deletes the feature [+participant] in the context of [+past] (i.e. the imperfect and aorist).

(14)
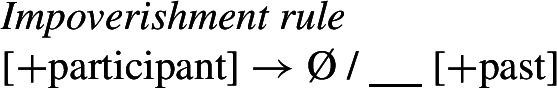

As Bobaljik points out, this rule can account for the apparent conspiracy between position classes II (≈ Tense) and III (≈ Agr). Since [+participant] is deleted on Agr, this bleeds insertion of the more specific exponent *-š* in (12a) and we therefore retreat to the more general 3rd singular marker *-še* in (12b).^4^ At the same time, deletion of [+participant] in Agr removes the feature mentioned in the contextual specification in (13a) that is needed for insertion of *-v* in the Tense head. This leads to the insertion of the more general zero exponent in (13b) and thus to the realization of the structure of a 2sg imperfect verb in (15).

(15)

Thus, the apparent virtue of a rule of referral analysis in deriving conspiracies in whole-word syncretism equally holds of an impoverishment account in which impoverishment may bleed the immediate and contextual specifications for realization.

Similar to Stump’s rule of referral, the impoverishment rule in (14) also enforces directional spreading of the 3rd form to a 2nd person context in both position classes. Furthermore, Bobaljik points out that the impoverishment account has the advantage of being more restrictive in this regard. A rule of referral could in principle also stipulate a take-over in the opposite direction (from 2nd to 3rd), whereas this would not be possible in an impoverishment analysis under the assumption that deletion of features can only derive a third person feature specification from a second person one, given the VIs in (12). It has therefore been argued that the DM approach imposes a directionality restriction on syncretism. As summarized by Bobaljik (2002: 64), ‘impoverishment rules […] embody the hypothesis that true syncretism […] will always be neutralizations towards lesser marked forms’.

On this view, a form A is less marked than another form B if A realizes fewer features of the context than B. As we have seen, an impoverishment approach to directional syncretism involves bleeding insertion of an otherwise more specific, i.e. more marked, exponent in favour of a less specific, and therefore less marked, form. This is the case for the (b)-entries in (12) and (13), which are inserted instead of the more specific (a)-forms, as is also the case for the Polish VIs in (7). The DM hypothesis is therefore that directional syncretism always involves spreading of less marked exponents. We will call this the *Unmarkedness Hypothesis* (16).

(16) *Unmarkedness Hypothesis (version 1)*
  If a feature set X takes the exponent associated with another feature set Y, then the feature specification of Y’s exponent is less marked than the feature specification of X’s exponent.

If this hypothesis can be maintained, then this would be an argument in favour of the DM approach to syncretism, as a more restrictive theory should be preferred over a more powerful alternative (see e.g. Noyer, 1998: 283; Bobaljik, 2002: 66–67; also see Kramer, 2016).

It has been argued, however, that the DM approach to syncretism, including the Unmarkedness Hypothesis, is empirically inadequate in light of cases of so-called *bidirectional syncretism* (Stump, 2001; Baerman, 2004; Baerman et al., 2005; Spencer, 2019). As will be discussed in more detail in Sect. 3, bidirectional syncretism involves two instances of directional syncretism involving the same paradigm cells. We have already seen an example of this from nominal declension in Latin in (1), repeated below in (17), where with some nouns such as ‘war’, the nominative takes the suffix associated with the accusative (*-um*), while in a different class of nouns, the nominative suffix *-us* is found in the accusative.

(17)
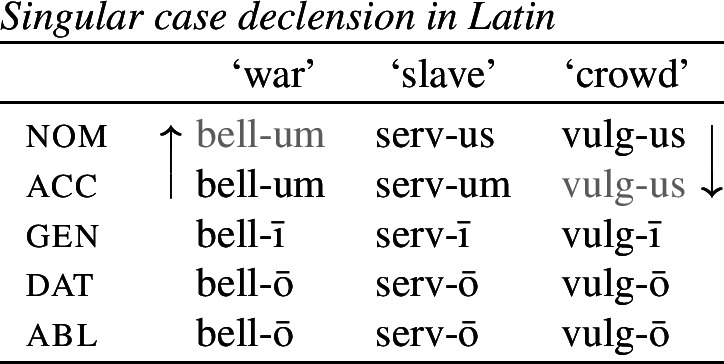

This bidirectional pattern poses a direct challenge to the idea that directional syncretism always involves neutralization to less marked forms. With nouns like ‘war’, the accusative suffix *-um* replaces the expected *-us* marker in the nominative, meaning that the accusative form must be the less marked of the two. However, this markedness asymmetry is directly contradicted by the nouns of the ‘crowd’-type, where the nominative form replaces the accusative. This would require contradictory specifications of the two exponents, with *-us* being more specific than *-um* for ‘war’ and the reverse being true for ‘crowd’.

It has therefore been argued that bidirectional syncretism falsifies the Unmarkedness Hypothesis and is thereby fatal for the DM approach to syncretism. For example, in discussing Noyer’s conjecture that the source of a directional syncretism (the spreading cell) is always less marked than its target, Stump (2001: 238) claims that ‘the very existence of bidirectional referrals is incompatible with Noyer’s conjecture’ and that it is therefore ‘empirically disconfirmed’. This sentiment is echoed in Spencer (2019: 25) who states that ‘what the DM model seems to exclude categorically are what [Baerman et al. (2005: 136)] call bidirectional syncretisms’. Furthermore, Baerman (2004: 816) has argued that, due to the fully overlapping distribution of exponents, the Latin paradigm in (17) ‘poses an insurmountable problem for symmetrical rules [i.e. underspecification]’, as DM would seem to require.

The claim that bidirectional syncretism poses a serious challenge to DM has not been taken up by those working in DM (though it has received passing mention as a potentially challenging phenomenon; e.g. Albright & Fuß, 2012: 269–271; Müller, 2013: 246–249; Kramer, 2016: 114, fn.22). As it stands, bidirectional patterns seem to be incompatible with the prevailing view of syncretism in DM and thereby undermine the supposed advantage of the more restricted impoverishment-based analysis over rules of referral.^5^

In what follows, we will revisit this debate and argue that, on closer inspection, even the most challenging instances of bidirectional syncretism are fully compatible with the approach to syncretism proposed by Noyer (1998), in which deletion may lead to insertion of unmarked feature values. What is more, we will show that this account of directional syncretism allows for a revised version of the Unmarkedness Hypothesis to be upheld, even in light of the challenge posed by bidirectional syncretism. Before we show this, we will discuss the phenomenon of bidirectional syncretism and its possible analyses in more detail.

## Bidirectional syncretism

The phenomenon of bidirectional syncretism was first identified by Stump (2001). Subsequently, Baerman (2004) proposed a distinction between two sub-types of bidirectional syncretism, *convergent* and *divergent*, as summarized abstractly in (18).

(18)

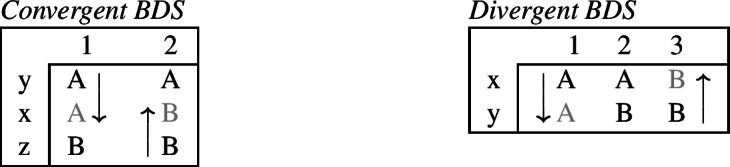

We will discuss each of these patterns in more detail below. As we will see, it is actually only the divergent one which poses a serious theoretical challenge to DM.

### Convergent bidirectional syncretism

The first type of BDS identified by Baerman (2004) is what he calls *convergent bidirectional syncretism*, as defined in (19).

(19) *Convergent bidirectional syncretism*
  There is a feature value *x* that takes the form associated with feature value *y* in some contexts, and in other contexts takes the form associated with feature value *z*.

A clear example of this pattern comes from case declension in Bonan. Both nouns and pronouns in the genitive are marked by *-ne*, while they take the suffix *-de* in the dative. In nouns, the accusative is syncretic with the genitive, while pronouns adopt exhibit a syncretism between accusative and dative. This is a case of convergent BDS according to (19) as the feature value acc takes the form associated with gen (*-ne*) in nouns and with dat (*-de*) in pronouns.^6^

(20)
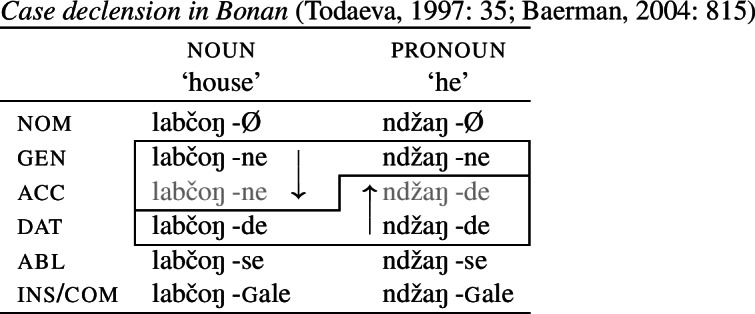

A very similar pattern of convergent BDS is found in the unrelated language Lak. Here, it is the ergative that borrows its forms from the absolutive and genitive in nouns and pronouns, respectively (21).

(21)
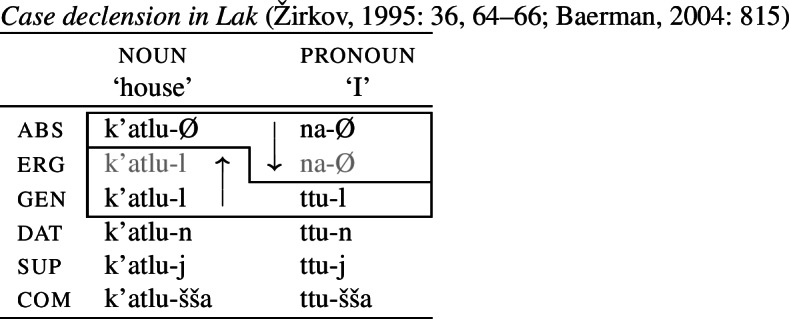

Another pattern discussed by Baerman (2004) is tense inflection in Gujarati. The 1st singular future marker is *-is̆*, while 3rd singular future verbs end in *-s̆e*. In the 2sg future, there is optionality (indicated here by two inflection classes). The 2sg may either take the form of the 1sg or the 3sg, giving rise to convergent bidirectionality across the two options.

(22)
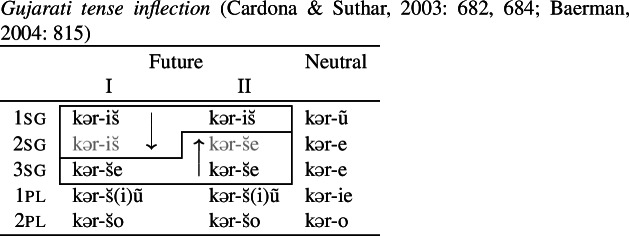

Another example of convergent BDS is mentioned in Pertsova (2011: 257). In Amele, 1st singular (and plural) inflection takes the form *-m*, whereas 3rd singular is marked by *-b* across all numbers. In the 2nd person, the 1st singular form is borrowed in the singular, while dual and plural take the form of the 3rd singular.

(23)
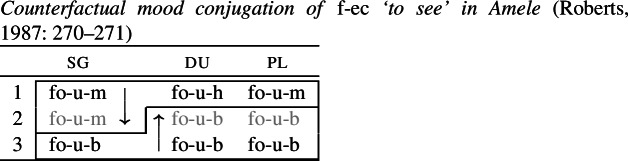

A slightly different instance of a convergent BDS pattern comes from Russian nominal inflection as also mentioned in Stump (1993, 2001) and Baerman (2004). In the plural (and also in the singular of class I which is not shown here), the accusative takes the form of the nominative with inanimate nouns. For animates, the accusative is marked with the exponent that is associated with the genitive (24). In contrast to the previous examples, the pattern repeats across the four inflection classes (and in the singular of class I) with different though partially overlapping exponents. It therefore constitutes a case of convergent bidirectional *metasyncretism* (Bobaljik, 2002; Harley, 2008).

(24)
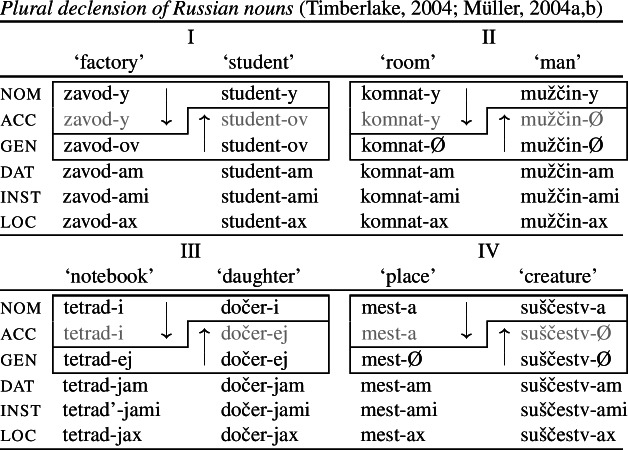

In all of these examples, there are two instances of directional syncretism, each indicated by an arrow. In each paradigm, the two take-overs are convergent because they have the same target cell (or dependent member in Stump’s 2001 terms). For example, in Bonan the accusative takes the form of the genitive for nouns, while the accusative takes the form of the dative for pronouns. In other words, both instances of directional spreading *converge* on the same feature value across the two contexts.

Baerman (2004) shows that convergent BDS can be analyzed with underspecification. The basic idea is to define overlapping classes, assigning each an ‘index’ such as X or Y. For convergent bidirectional syncretism in Bonan (20), X would be defined as the natural class corresponding to genitive and accusative (25a), while Y stands for the unification of dative and accusative (25b). These indices are then mapped to the corresponding forms by the realization rules in (26)[a–b]. The overlapping domains of X and Y mean that both *-ne* and *-de* should be equally possible forms for the accusative. Baerman (2004) argues that this indeterminacy can be resolved by the two ordering statements in (27). In each of the respective contexts (noun vs. pronoun), a different form will take precedence, thereby yielding the pattern of convergent bidirectional syncretism.

(25)

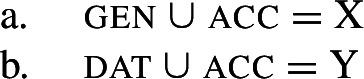

(26)

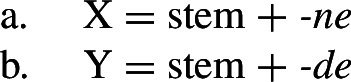

(27)

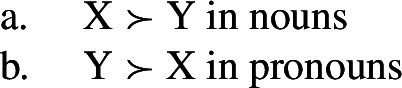

Harley (2008) proposes a very similar analysis of Bonan couched in Distributed Morphology. Instead of Baerman’s (2004) indices, Harley decomposes case values into binary sub-features. The decomposition of the relevant cases (genitive, accusative, and dative) is given in (28). On this decomposition, genitive and accusative share the feature [+structural], whereas dative and accusative have the common feature [+dependent]. These features form overlapping natural classes in a way similar to Baerman’s indices (X = [+structural] and Y = [+dependent]).

(28)
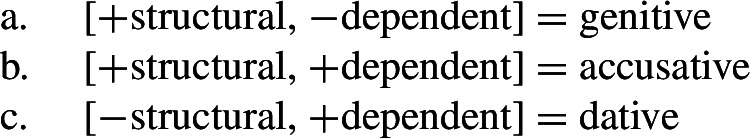

Harley proposes the following Vocabulary Items for the case forms:

(29)
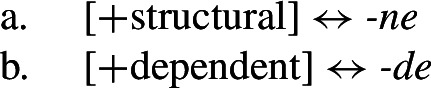

Due to the underspecification of these VIs, both of the exponents in (29) will be equally good candidates for insertion in the accusative, given the Subset Principle. To resolve this, Harley proposes the impoverishment rule for pronouns in (30) which deletes [+structural].^7^

(30)
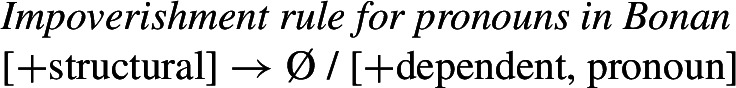

This means that although both *-ne* and *-de* are compatible markers for the accusative with pronouns, insertion of *-ne* is bled by the impoverishment rule, leading to insertion of *-de* (31).

(31)
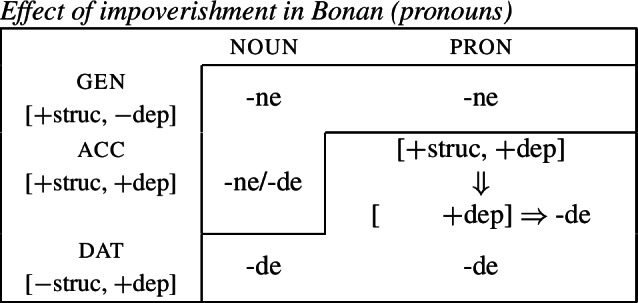

This still leaves us with an indeterminacy for the accusative in nouns. Here, Harley takes a similar approach to Baerman in proposing a feature hierarchy [±structural] ≻ [±dependent], which leads to a preference for insertion of exponents realizing [±structural] (*-ne*). Though as Kramer (2016: 114, fn. 22) points out with reference to Stump (2001: 281, fn. 3), the reliance on a feature hierarchy here might undermine the restrictiveness of this type of approach. Instead, we propose that it is equally possible to adopt the second impoverishment rule in (32) that deletes [+dependent] in the context of nouns.

(32)
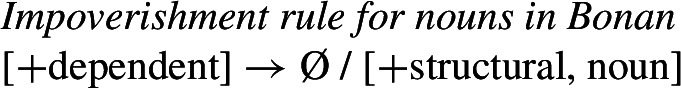

This bleeds the insertion context for *-de*, thereby leading to the insertion of *-ne* in accusative nouns (33).

(33)
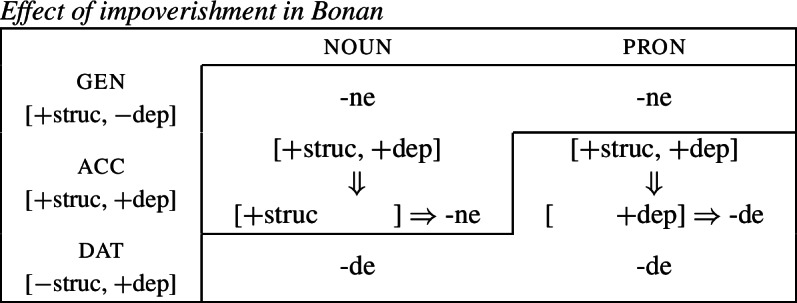

This serves to highlight that, at least on an implementational level, convergent bidirectional syncretism does not pose a significant challenge for an approach which derives syncretic patterns through underspecification plus impoverishment.

It does, however, raise the question of whether this analysis conforms to the *Unmarkedness Hypothesis* (16), which states that syncretism involves insertion of a relatively ‘less marked’ form. In this regard, ‘less marked’ typically translates to ‘less specific’, but both of the VIs in (29) is are equally specific, i.e. both realize a single feature of the insertion context. For this reason, it is unclear whether this analysis is strictly speaking compatible with this formulation of the Unmarkedness Hypothesis. While one solution could be to reformulate the hypothesis to say that directional syncretism may not increase markedness, the reformulation of the *Unmarkedness Hypothesis* that we will propose in Sect. 4.3, which is stated over contexts rather than forms, does not suffer from the problem mentioned above.

### Divergent bidirectional syncretism

The second pattern of bidirectional syncretism identified by Baerman (2004) is called *divergent bidirectional syncretism (divergent BDS)*, as defined in (34).

(34) *Divergent bidirectional syncretism*
  There is a feature value *x* that takes the form associated with feature value *y* in some contexts, while in other contexts *y* takes the form associated with *x*.

Below, we present four illustrative examples of this pattern. Further potential examples include (pro)nominal inflection in Diyari discussed in Baerman (2004) (though see Bierkandt, 2006; Müller, 2008: 225, fn.14 for important qualifications) and verb conjugation in Romanian discussed in Stump (2001). We discuss the latter in detail in Sect. 4.4.

The first example is taken from the nouns of the so-called second declension in Latin. As Baerman (2004) points out, this paradigm exhibits divergent BDS. In nouns of what we call class II (consisting of masculine nouns), the nominative suffix is *-us* and the accusative suffix is *-um*. In class I, corresponding to most neuter nouns, the nominative takes the form of the accusative (*-um*). This take-over goes in the opposite direction with a sub-class of neuter nouns that we assign to class III, e.g. *vulgus* (‘crowd’), *vīrus* (‘poison’), *pelagus* (‘sea’). We therefore have bidirectionality: spreading from acc to nom in class I nouns and spreading in the opposite direction from nom to acc in class III nouns.

(35)
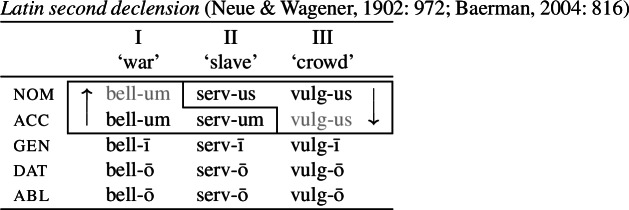

A further example discussed in Baerman (2004) comes from Classical Arabic nominal declension. As we see in (36), the triptotic pattern, which can be taken as the default pattern, shows a three-way case distinction, *-u* for the nominative, *-i* for the genitive and *-a* for the accusative. In the diptotic pattern, which is only found with indefinite nouns, the genitive takes the *-a* suffix of the accusative. For plural nouns like ‘believers’, however, it is the accusative that borrows the form of the genitive (*-i(ː)*).

(36)
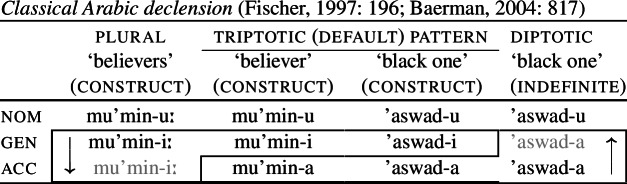

Another example of divergent BDS can be found in Old Icelandic nominal declension, as discussed by Stump (1993) (citing Heusler, 1962: 64f., Noreen, 1923: 260ff., Gordon, 1957: 285). Here, we also see directional syncretism within different inflection classes for nouns. Class B nouns in nom/acc forms take a null case suffix, while the dative is marked by *-o* in this class. In nouns belonging to Class A, we find that the case ending in the dative takes the form of the syncretised nom/acc cells. In Class C, however, the accusative takes the form of the dative. Again, this is a case of divergent BDS.

(37)

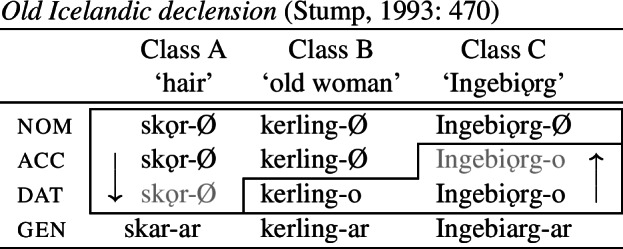

As with convergent bidirectional syncretism, all of these examples involve two instances of directional syncretism (indicated by the arrows). Unlike the convergent patterns, however, the directional syncretisms here have distinct targets.^8^ In the Latin example, for instance, the nominative takes the form of the accusative (*-um*) for one class of nouns whereas the accusative takes the form of the nominative (*-us*) for another class of nouns.

Let us now consider whether it is possible for an impoverishment-based approach to account for the divergent pattern. For the purposes of illustration, we will focus on the Latin paradigm. Starting from the distribution of the two markers in class II, we could first take them to be fully specified nominative and accusative markers, respectively. Taking the Latin case values to be decomposed into sub-features along the lines of Halle (1997), nominative corresponds to [+superior, +structural] and accusative is decomposed into [−superior, +structural].^9^ The entries for the relevant Latin exponents are given in (38).

(38)
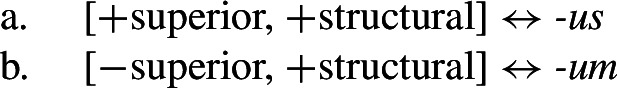

We would then have to assume an impoverishment rule like (39) to bleed insertion of *-us* in the nominative of class I.

(39)
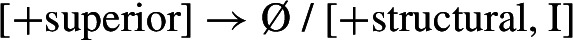

Consequently, the accusative marker would have to be underspecified such that it fits both accusative and nominative contexts. We achieve this by associating it with the sub-feature that both cases have in common, namely [+structural] (40b).

(40)
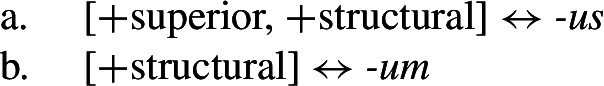

Impoverishment successfully bleeds insertion of the expected nominative marker in (40a), thereby yielding a retreat to the more general exponent in (40b). This is shown in (41).

(41)
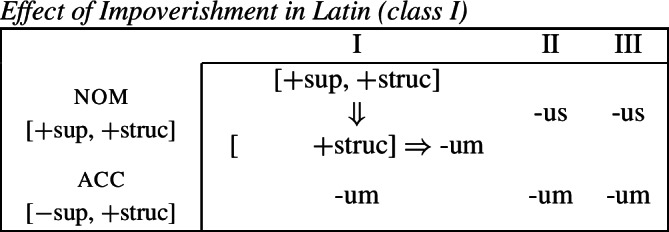

With this in place, we now need to force *-us* to appear in the class III accusative context. Given the marker specifications in (40), *-us* is not eligible for insertion as it is specified for [+superior]. This feature clashes with the [−superior] of the accusative context. To enable insertion of *-us* in the accusative, it must also be underspecified for [±superior], as in (42).

(42)
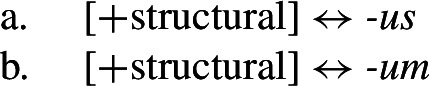

The problem that this approach faces now becomes apparent. Irrespective of how we derive the corner cells, the exponents *-us* and *-um* require a fully overlapping distribution, as pointed out by Baerman (2004). The consequence of this is complete indeterminacy throughout the paradigm (43).

(43)
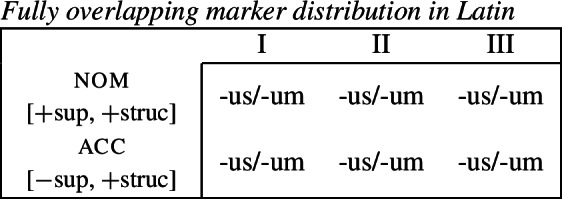

While an indeterminacy of marker selection is not per se a problem and has been argued to variably give rise to optionality (Hein, 2008; Driemel, 2018; Davis, 2021) or ineffability (Coon & Keine, 2021), we observe no such effects in the Latin paradigm. This situation also differs from the partially overlapping distribution that we saw in Bonan and there seems to pose a serious challenge for the DM view of syncretism.^10^

It is important to note that the indeterminacy problem only arises with bidirectional paradigms in which there is spreading of two distinct markers. Consider the singular declension of Latvian nouns in (44). The first suffix is the theme vowel which is irrelevant for present purposes. The second suffix expresses a combination of case and number. For most Class A singular nouns, the case ending for the nominative is *-s*, while genitives take *-a*. For some singular nouns of Class A, exemplified by *tirg* (‘market’) in (44), the genitive takes the form of the nominative (*-s*) rather than the expected *-a* case suffix. In Class B, the expected nominative singular marker is *-Ø*, while genitives take *-s*. With certain class B nouns such as *gov* (‘cow’), the nominative has the same form as the genitive in this class (*-s*).

(44)
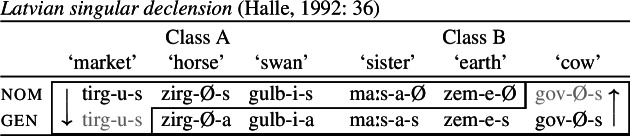

While this paradigm exhibits a pattern of divergent BDS with nominative and genitive forms spreading in distinct contexts, there is an important difference to the cases discussed above. In Latvian, bidirectionality involves the same suffix *-s*, which must be assumed to have a more general distribution, i.e. at least underspecified for the nom/gen distinction (Halle, 1992: 40 treats it as completely underspecified). Thus, both of the directional syncretisms in (44) can be treated as a retreat to the general case. Since it is the same marker spreading in each case, there is no indeterminacy problem associated with an overlapping distribution.

Returning to bidirectional syncretism involving two exponents, a possible solution to the indeterminacy problem faced by an impoverishment-based analysis could be to try to reanalyze all putative examples of divergent BDS as convergent (see e.g. Müller, 2008: 210). For example, we could try to reanalyze Latin as convergent BDS if we treat the nominative and accusative contexts not as case syncretism but as syncretism for inflection class. This alternative view is presented in (45).

(45)
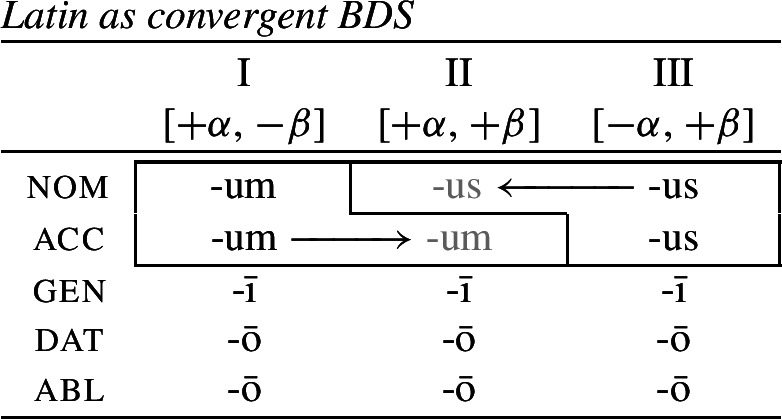

However, this would require creating natural classes of inflectional classes by means of feature decomposition (see e.g. Müller, 2004a,b; Alexiadou & Müller, 2008). We would therefore treat *-um* and *-us* as class markers rather than case markers (46).

(46)
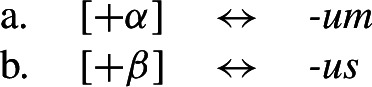

In order to derive this convergent directional spreading, we would need the two impoverishment rules in (47), similar to the analysis of Bonan.

(47)
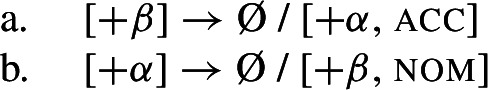

Aside from the issue of now viewing the Latin paradigm as involving a mix of syncretism for case and inflection class, the main challenge here seems to relate to markedness. While markedness for traditional grammatical categories, such as person, number or case, has been argued to have an independent cross-linguistic grounding (e.g. Greenberg, 1966; Silverstein, 1976), similar motivation is much more difficult to establish for relative markedness distinctions between arbitrary language-specific inflection classes (though see Oltra Massuet, 1999 and Noyer, 2005 for ideas in this direction).

We therefore agree with Baerman (2004) that divergent bidirectional syncretism poses a genuine challenge for underspecification plus impoverishment-based approaches to syncretism.^11^ By contrast, rules of referral can readily capture divergent bidirectional syncretism. The forms *-us* and *-um* would be straightforward nominative and accusative exponents, as in the realization rules in (48).

(48)
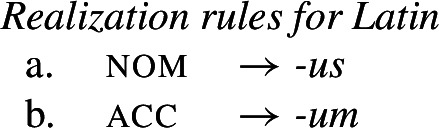

These are then superseded by the two rules of referral in (49), which simply state each individual directional syncretism in the bidirectional pattern.

(49)
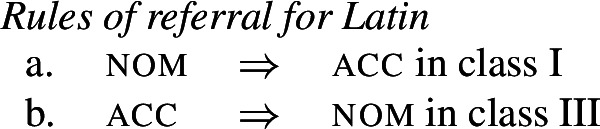

In conclusion, divergent BDS does indeed seem to pose a serious challenge for the DM approach to syncretism, given the fully overlapping distribution that underspecification would require. Furthermore, it has also been argued to prove fatal for the idea that directional syncretism can be constrained by markedness (Stump, 2001; Baerman et al., 2005), since it clearly entails a contradiction in the relative markedness of the two feature values.

In the following section, however, we will argue that divergent BDS ceases to be problematic once we adopt Noyer’s (1998) enriched theory of impoverishment, whereby unmarked feature values may be inserted as the result of deletion.

## Impoverishment and feature insertion

In this section, we will present Noyer’s (1998) proposal that impoverishment may lead to insertion of an unmarked feature value (also see Harbour, 2003; Calabrese, 2011; Arregi & Nevins, 2012). We will recap Noyer’s motivation for this innovation based on the complex interacting patterns of directional syncretism in Nimboran. Subsequently, we will go on to show that this approach affords us the flexibility to accommodate divergent BDS in a DM analysis, while still maintaining a version of the Unmarkedness Hypothesis.

### Directional syncretism in Nimboran

The view of impoverishment that will provide a solution to the challenge posed by divergent BDS originates from Noyer’s (1998) analysis of directional syncretism in Nimboran, a Papuan language of New Guinea, as described in the grammar by Anceaux (1965) and given a lexicalist analysis in Inkelas (1993).

There are several ways in which morphological realization is determined by the number of the subject in Nimboran. With a 1st person singular subject (50a), the stem takes its ‘A-form’ and there is no other dedicated number morpheme. For 1st person (exclusive) dual subjects, the stem takes its ‘B-form’ and the additional suffix *-k* (50b). Finally, when the subject is 1st person exclusive plural, the stem is realized in its ‘C-form’ and there is an additional suffix that we represent as a superscript ^*-i*^ following Noyer (1998) and Keine (2013) (Inkelas, 1993 uses 〈i〉). This suffix is a floating segment that palatalizes neighbouring consonants. For example, the word in (50c) is realized on the surface as *ŋgedóidiu* (Inkelas, 1993: 584).

(50)
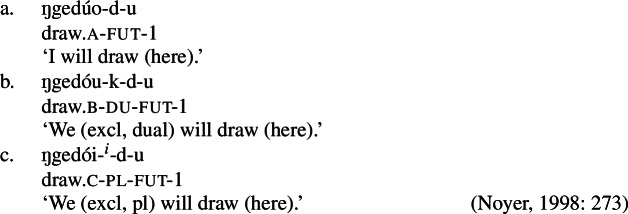

The distribution of the stem forms and subject agreement affixes is summarized in (51).^12^ We can identify three relevant morphological slots here: the verb stem (I), the number agreement suffix (II) and the person agreement suffix (III). Here, we see that the person agreement markers in slot III also show gender distinctions in the 3rd person. The *-am* suffix marks a 3rd person masculine subject in the singular and dual, while the suffix *-um* marks a 3rd person non-masculine subject (e.g. feminine or inanimate) in the singular and dual. This distinction is neutralized in the plural, where the suffix *-am* marks both masculines and non-masculines.

(51)
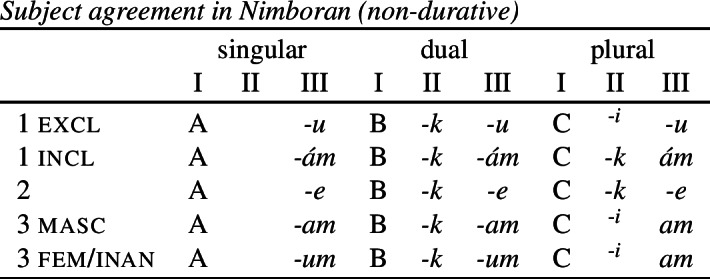

We already see an instance of directional syncretism in (51), namely the spreading of the 3rd person masculine marker *-am* in the plural. The paradigm in (51) also shows that the dual marker *-k* in slot II spreads to the plural in the 1st person inclusive and the 2nd person.

We also find directional spreading in what Noyer calls the ‘special environment’, durative aspect. In the durative, singular subjects trigger the B-form of the stem (52a), which is typically viewed as the default stem. Furthermore, the distinction between dual and plural is neutralized completely in durative contexts. As such, the form in (52b) containing the C-form of the stem and the number suffix ^*-i*^ can be interpreted as having either a dual or a plural subject.

(52)
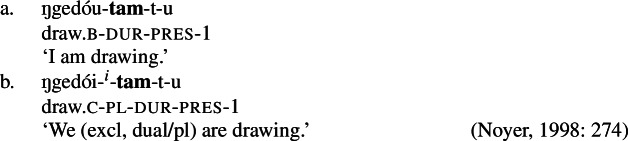

The distribution of stem forms and the relevant agreement affixes for durative verbs is given in (53).

(53)
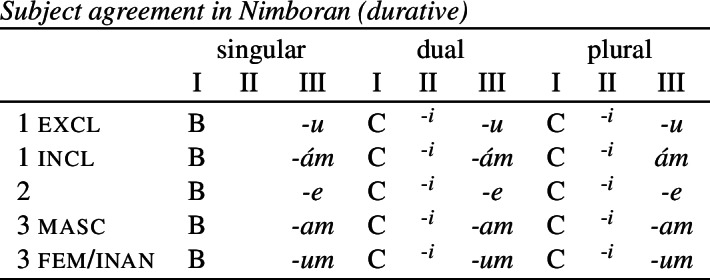

The relevant generalizations about directional syncretism in Nimboran subject agreement morphology can be summarized as follows:

(54)
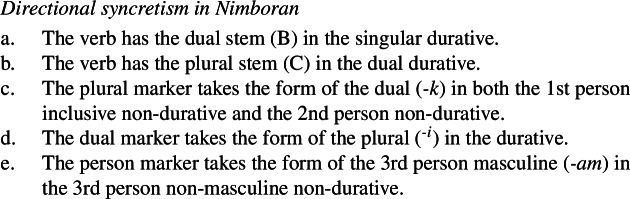

We also present these take-overs visually for the stem forms (55), the number affixes (56), and the gender affixes (57). We also provide the relevant feature decomposition that Noyer assumes for number [±sg, ±pl], number [±1, ±2] and gender [±masc].^13^

(55)

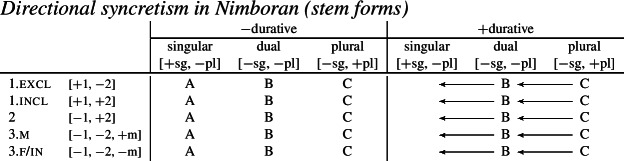

(56)

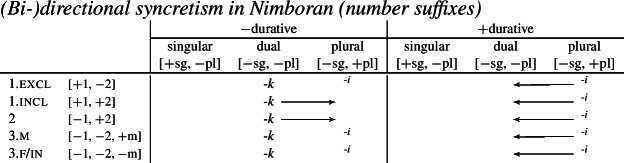

(57)

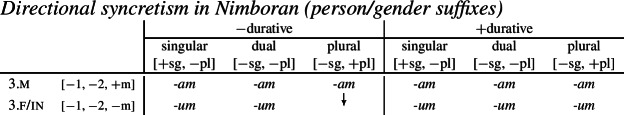

Let us start with the directional syncretism for the number suffixes in (56). Noyer assumes that *-k* is the dual marker and ^*-i*^ is the plural marker. In [−durative] contexts, the dual marker spreads to the plural in both 1st person inclusive and second person, a natural class we can define with the decomposed feature [+2]. As pointed out by Keine (2013: 218), this syncretism is bidirectional: *-k* spreads from dual to plural in non-durative [+2] contexts, while ^*-i*^ spreads from the plural to the dual in the durative. On an underspecification approach, both the markers for dual and plural would have be specified to realize only [−sg] in order to be eligible for insertion into both dual and plural contexts. However, this raises a serious problem, as there is no obvious way to have one marker block the other. This is essentially the same problem we faced for Latin, precisely because this bidirectional number syncretism in Nimboran is also divergent.

To solve this problem, Noyer proposes the following. First, he assumes the Vocabulary Items in (58), where the dual marker realizes [−sg] and the plural marker realizes [+pl]. In order to achieve the directional spreading from dual to plural in [+2, −durative] contexts, Noyer proposes the impoverishment rule in (59) which deletes [+pl]. This ensures that insertion of the plural exponent *-i* (58b) is bled and the dual suffix (58a) surfaces instead.^14^

(58)

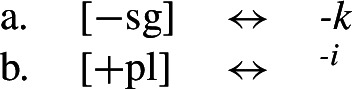

(59)

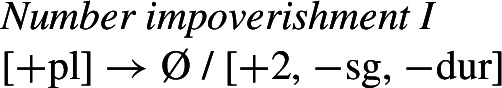

In the [+durative] cells, the plural marker ^*-i*^ spreads to the dual cells. This presents a technical problem, however, as this directional spreading cannot be derived by impoverishment alone, given the marker specifications in (58). As we have mentioned, impoverishment typically only allows for a ‘retreat to the general case’ by means of feature deletion. In other words, a less specific, yet still compatible, marker can be inserted by virtue of an impoverishment rule. A marker specified for [+pl], however, will never be eligible for insertion in a dual context ([−sg, −pl]) due to the Subset Principle. The directional syncretism in [+durative] contexts must therefore be derived by somehow changing the specification of the dual cell from [−sg, −pl] to [−sg, +pl] so that the exponent in (58b) may be inserted.

This is where Noyer’s technical innovation comes in. He proposes that deletion of a particular feature may lead to insertion of a default value. Noyer proposes that such feature insertion is the result of ‘redundancy rules’ that fill in the contextually unmarked values in a particular feature combination (though Embick & Noyer, 2007: 313 simply refer to these as ‘markedness rules’). In order to derive the problematic spreading of plural ^*-i*^ to dual contexts, he proposes the impoverishment rule in (60a) that deletes the [−pl] feature of the dual in the durative. This allows for the redundancy rule in (60b) to insert the [+pl] feature in its place.

(60)
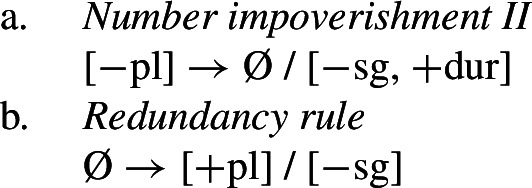

The derivation for this is given in (61). Here, it is possible to turn a dual context into a plural one using the combination of an impoverishment rule that feeds a redundancy rule inserting [+pl] in the context of [−sg].

(61)
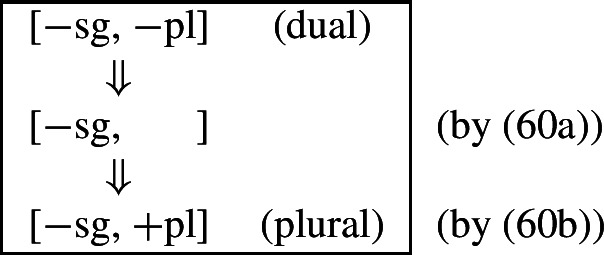

An important part of Noyer’s proposal is that it envisages an inherent restriction on this kind of feature insertion. In contrast to rules of referral, redundancy rules may only ever insert a feature whose value is unmarked relative to another sub-feature that makes up that context. Markedness is therefore determined contextually (Nevins, 2011).

To see this more clearly, consider the universal markedness hierarchy for number assumed by Noyer, in which dual is more marked than plural, and plural is more marked than singular:

(62)
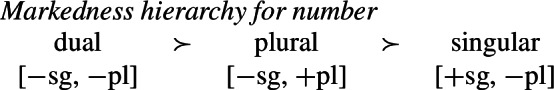

Here, the contextual markedness relations can be read off the hierarchy directly. Both dual and plural share the sub-feature [−sg]. Since dual is more marked than plural according to the hierarchy, the negative value for [±pl] is more marked than the positive one in the context of [−sg]. For this reason, only the redundancy rule in (61b) is possible. A comparable rule inserting [−pl] in the context of [−sg] is ruled out as it would constitute insertion of the contextually-marked value. In other words, a plural context may not be transformed into a dual context. In principle, one could envisage precisely such an analysis for the directional spreading of *-k* in the non-durative as involving a change from [+pl] to [−pl], but, as this kind of directional syncretism by feature-changing is markedness increasing, it must instead be analyzed as an instance of retreat to the general case by means of feature deletion. This distinction will be crucial for our analysis of divergent BDS in Latin.

Noyer’s assumption of a general neutralization of dual/plural in the durative has wide reaching consequences beyond the bidirectional number syncretism. Let us now consider the change in stem forms in the durative. Recall that, in the non-durative, the A-stem is found with singular subjects, the B-stem occurs with dual subjects and the C-stem marks plural (55). Noyer proposes the VIs for root allomorphs in (63).^15^

(63)
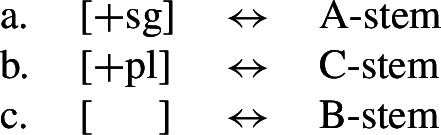

In the durative, the B-form spreads to the singular and the C-form spreads to the dual. The takeover of the ‘plural’ stem form in the [+durative] is entirely expected as a result of the rules in (60). After they apply, the durative dual contexts are featurally identical to plural contexts. For this reason, the VI in (63b) takes precedence over the elsewhere B-form in (63c).

Insertion of the A-form is bled in the durative, where we instead see a takeover of the B-stem. This is straightforwardly captured as a retreat to the general case enforced by impoverishment of the singular. For reasons that will become clear in a moment, Noyer actually assumes that all [±sg] features are deleted in the durative by virtue of a third number impoverishment rule in (64).

(64)

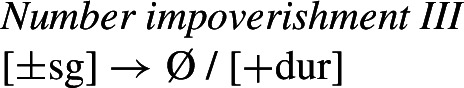

While this rule captures the directional spreading of the B form to the singular, it also has another effect regarding gender syncretism. Recall that gender distinctions are neutralized in the 3rd person plural non-durative (57). While the masculine forms are marked with *-am* and feminine/inanimates with *-um*, both are marked with *-am* in the non-durative plural. This can be viewed as directional spreading of the masculine marker. Noyer analyzes this as a retreat to the general case by assuming the VIs in (65) for position III, where *-um* is specified for [−masc] contexts and *-am* is the elsewhere marker.^16^

(65)
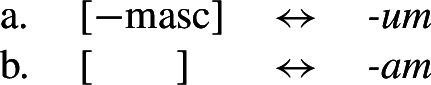

In order to derive the neutralization of a gender contrast in the 3rd person non-durative, we need at least the impoverishment rule in (67) which deletes [−masc] in the 3rd person plural.

(66)
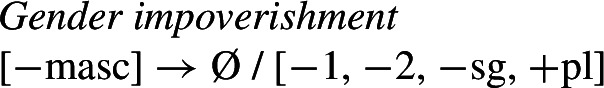

What is interesting is that the context for this impoverishment rule does not mention the [±durative] feature, despite the fact that this rule only applies in [−durative] contexts. The reason for this is that the gender impoverishment rule is bled by the independently-motivated number impoverishment rule in (64), which deletes the [−sg] feature mentioned in the contextual specification of (66). Thus, Noyer’s analysis provides a deeper explanation for why gender distinctions are no longer neutralized in the durative. The neutralization of [±sg] distinctions that is responsible for the stem changes in the durative also bleeds the application of any rule sensitive to this feature.

To summarize, Noyer’s approach requires four impoverishment rules (three for number and one for gender). These are listed below in (67). The rule in (67b) feeds the redundancy rule in (67c), which together constitute a kind of feature-changing rule.

(67)
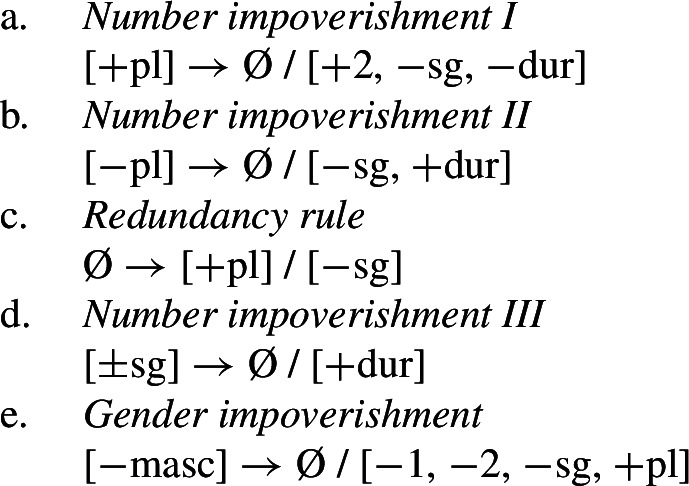

Recall the five instances of directional syncretism that we identified in (54), repeated below.

(68)
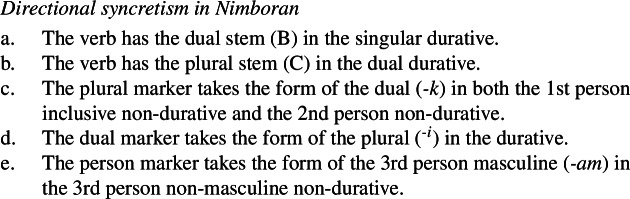

The spreading of the B-stem to the singular in the durative mentioned in (68a) is captured by *Number impoverishment III*. *Number impoverishment II* in conjunction with the redundancy rule accounts for both (68b) and (68d) as both involve the same shift from [−pl] to [+pl] in the durative. *Number impoverishment I* accounts for (68c) as deletion of [+pl] bleeds the insertion of the expected plural suffix *-k*. Finally, the gender syncretism in the non-durative (69e) is accounted for by *Gender impoverishment*. Furthermore, the fact that this latter rule does not apply in the durative is captured by the fact that *Number impoverishment III* bleeds its application context.

Thus, Noyer’s account provides an insightful account of the various directional syncretisms in Nimboran verbal inflection. This analysis allows for a deeper relation between them. Some of the syncretisms are derived by one and the same rule, e.g. (68b) and (68d), whereas the rule for deriving one take-over (69a) is responsible for blocking an apparently unrelated instance of spreading (68b) in the same context. Noyer’s account sheds light on the deeper system behind the syncretism: Nimboran neutralizes certain number distinctions, namely [±pl] and [±sg], in the durative. In order to account for spreading of plural markers to the dual contexts, Noyer motivates a new theoretical proposal in terms of insertion of unmarked feature values as a consequence of impoverishment. As we will show in the following section, his solution is readily applicable to the problematic cases of divergent BDS that we have seen.

### Divergent bidirectional syncretism revisited

With this in place, we can now return to divergent BDS in Latin, repeated in (69), as a representative example, we will see how Noyer’s approach to impoverishment will allow us to avoid the previously discussed problems.

(69)
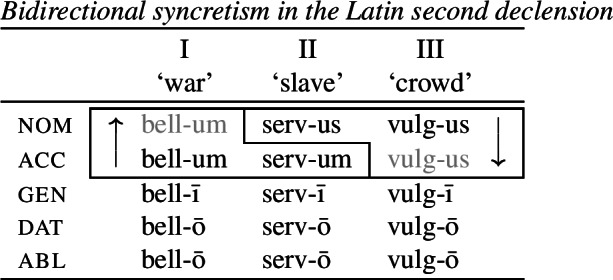

We start again by taking *-us* and *-um* to be fully specified nominative and accusative markers, respectively (70).

(70)
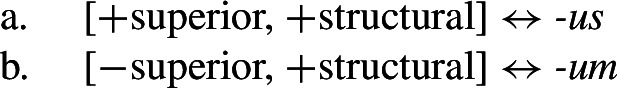

Following work on the case hierarchy (Blake, 2001; Caha, 2009; McFadden, 2018; Smith et al., 2019), we take the nominative to be less marked than the accusative. This in turn means that [+superior] is the contextually unmarked value of [±superior] in the presence of [+structural]. This can be seen in the decomposed hierarchy in (71).

(71)
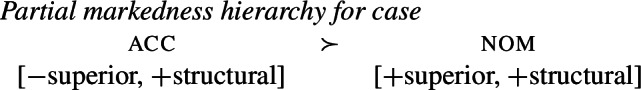

Focussing on the take-over of the accusative by the nominative in the class III first, we can adopt the impoverishment rule in (72), which deletes [−superior] in the accusative of class III.

(72)
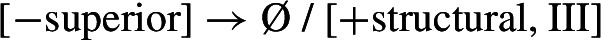

Since [−superior] is the marked value in this context, following Noyer’s proposal, the redundancy rule in (73) inserts the unmarked [+superior] feature once [−superior] is deleted.

(73)
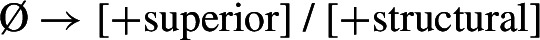

Effectively, this allows us to turn the accusative context into a fully specified nominative context, thereby bleeding the insertion of the otherwise expected accusative marker *-um* in favour of the nominative marker *-us* (74).

(74)

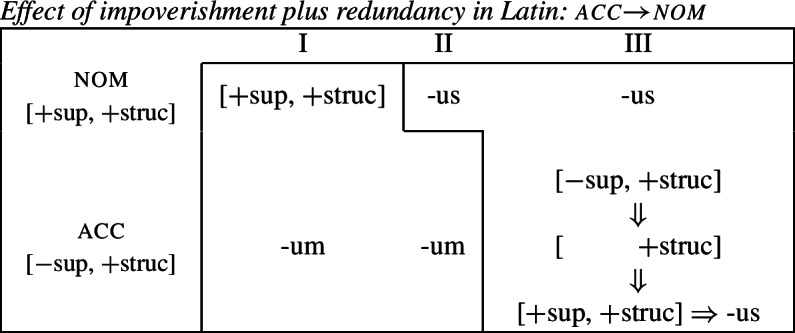

Turning to the take-over in class I, we need to bleed the insertion of the expected nominative form *-us*. This can be achieved by the impoverishment rule in (75) which deletes [+superior] in the context of [+structural] in class I.

(75)
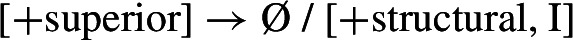

This is a case of deletion of a feature with a contextually unmarked value. It is important to note that Noyer (1998) assumes that, once deleted, an unmarked value may not be reinserted by a redundancy rule. Doing so would lead to a vacuous ‘Duke-of-York’ derivation (Pullum, 1976). Therefore, an application of the impoverishment rule (75) results in an underdetermined case context defined only as [+structural]. If we revise our marker specifications such that *-um* is underspecified for exactly this feature (76b), then the application of the impoverishment rule (75) triggers a retreat to the general case.

(76)
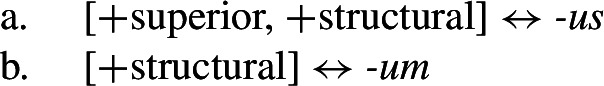

As can be seen in (77), this bleeds insertion of the otherwise expected exponent *-us* and thereby feeds the insertion of the less specific exponent *-um*.

(77)
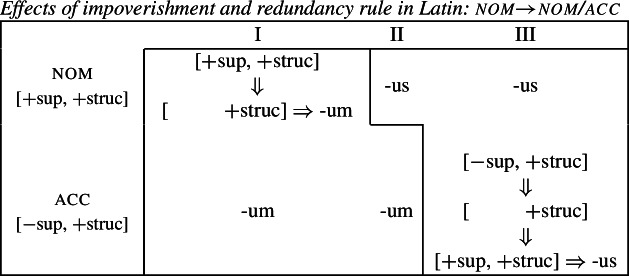

We therefore successfully derive the formerly challenging case of divergent BDS precisely because Noyer’s system allows for impoverishment to have two distinct outcomes. If the contextually-marked feature is deleted, as in class III accusative, then a redundancy rule will insert the unmarked feature. If the contextually-unmarked feature is deleted, as in the nominative in class I, then the redundancy rule may not re-insert this value. For this reason, the directional syncretism in class III involves a genuine feature change, while the spreading from accusative to nominative in class I is a retreat to the general case involving simple deletion. This then allows one to avoid the indeterminacy problem identified by Baerman (2004) for divergent BDS since the markers are no longer required to have fully overlapping underlying distribution.

### Consequences for the unmarkedness hypothesis

The previous section showed that divergent BDS can be readily analyzed with Noyer’s system, but recall that it was also shown to be in conflict with the hypothesis that directional syncretism is subject to a markedness restriction, since the bidirectional nature of the pattern will necessarily lead to a contradiction with regard to markedness.

The relevant question at this point is whether the analysis developed in the preceding section is still compatible with the Unmarkedness Hypothesis as stated in (16), repeated below.

(78) *Unmarkedness Hypothesis (version 1)*
  If a feature set X takes the exponent associated with another feature set Y, then the feature specification of Y’s exponent is less marked than the feature specification of X’s exponent.

The answer is clearly negative. An inspection of the entries for the Latin case suffixes that we assumed in (76) reveals that *-um* is less specific and should therefore count as less marked than *-us* (79).

(79)
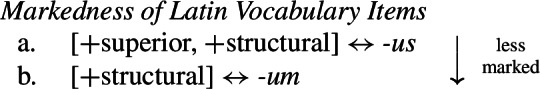

Nevertheless, the more marked exponent *-us* spreads into the accusative (blocking less marked *-um*) in class III. This clearly contradicts the Unmarkedness Hypothesis in its formulation in (16) in terms of exponents.

This should be a cause for concern because it has been argued that a potential virtue of the impoverishment-based approach over rules of referral is that directional syncretism is subject to an inherent markedness restriction (Bobaljik, 2002). The analysis developed for Latin is compatible with a slightly different perspective on markedness reduction, however. In fact, Noyer (1998) originally suggested a different view of markedness as holding not of exponents themselves, but rather insertion contexts: ‘Impoverishment-plus-Insertion will always move from a more marked to a less marked state’ (Noyer, 1998: 282). If we adopt this view, we can formulate the alternative version of the Unmarkedness Hypothesis in (80).

(80) *Unmarkedness Hypothesis (version 2)*
  If a feature set X takes the exponent associated with another feature set Y, then there must be a reduction in the markedness of the feature specification of X.

Under this view, our account of divergent bidirectional syncretism is consistent with the Unmarkedness Hypothesis in the following way. Since markedness no longer holds over exponents, but rather contexts, impoverishment can have two distinct outcomes: either a contextually-marked feature is deleted, leading to insertion of the unmarked value, or deletion targets a contextually-unmarked feature, leading to an impoverished or underdetermined feature combination. For this reason, this system actually distinguishes three possible levels of markedness. To see this, consider the expanded version of the partial case hierarchy in (81) containing nominative and accusative that we presented in (71).

(81)
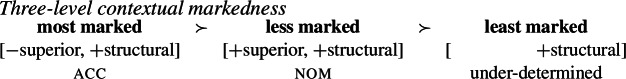

As both cases share the feature [+structural], this determines ‘+’ to be the unmarked value of [±superior] in the context of [+structural] since [+superior, +structural] make up the less-marked nominative. The marked value for [±superior] is ‘−’ since [−superior, +structural] together create a more marked accusative configuration. Given the fact that we also have impoverishment, it is possible to have a feature combination that lacks [±superior] altogether. We suggest that this combination is even less marked than the unmarked feature combination that corresponds to the nominative. This gives rise to the three levels of markedness given in (81): most marked, less marked and least marked.

Returning to the analysis of divergent BDS in Latin, each directional syncretism involves a reduction of contextual markedness of the underlying feature set. In the spreading of *-us* into the accusative in class III markedness is reduced from [−superior, +structural] to [+superior, +structural], i.e. moving from the most marked accusative specification into a less marked nominative one. The take-over of *-um* into the nominative of class I, on the other hand, involves a markedness reduction in the insertion context from [+superior, +structural] to [+structural], a canonical ‘retreat to the general case’ scenario, thereby turning an underlying nominative specification into an underspecified case context.

In conclusion, not only have we shown that it is possible to derive divergent BDS with impoverishment and underspecification, it is also possible to uphold a revised version of the Unmarkedness Hypothesis (stated over contexts rather than exponents) in light of this apparent counterexample to it (*pace* Stump, 2001).

At this juncture, one might wonder whether what we have said about a feature manipulation account such as impoverishment in DM could not also simply be transferred over to an approach employing a directional rule or rule of referral. In other words, could we not simply state that a rule of referral may only point to a less-marked feature configuration? We think that the answer here is negative. The inherent markedness contradiction in divergent BDS will still ultimately pose a problem since it can only be avoided, as we have shown, by referring to an underspecified context that corresponds neither to nominative or accusative. As such, the feature set containing just [+structural] does not correspond to any cell in the paradigm that could be targeted by a rule of referral in the traditional sense (e.g. Zwicky, 1985; Corbett & Fraser, 1993; Stump, 2001; Baerman, 2004).^17^

What we instead require is an approach to directional syncretism, which can refer to underspecified morphosyntactic contexts. This can be achieved by feature deletion and restricted insertion in Noyer-style DM analysis discussed here. Furthermore, it also seems to be possible in the second iteration of PFM in Stump (2016) which envisages an additional level of representation (the *form paradigm*), the mapping to which can involve the deletion/omission of features (much like the intermediate PF representations post-impoverishment in DM). If the markedness-decreasing stipulation that we have assumed also holds for the mapping to the form paradigm in PFM2, then the central insight of our analysis appears to be transferable. What both of these frameworks (DM and PFM2) have in common is that they do not derive directional syncretism by means of traditional rules of referral, but rather with manipulation of feature sets prior to realization.

### Bidirectional syncretism in Romanian: a remaining challenge?

So far, we have argued that patterns of bidirectional syncretism can be handled in a Noyer-style system in a way that is compatible with the view that syncretism is strictly markedness reducing. In this section, we will address a remaining case of bidirectional syncretism that appears to still pose a challenge to our approach.

Consider the present indicative verbal conjugation of Romanian, illustrated by the eight verbs in (82), which Stump (2001) claims exhibits bidirectional syncretism. (Note that the decomposition into inflection classes is not taken from Stump. We return to this momentarily.)

(82)

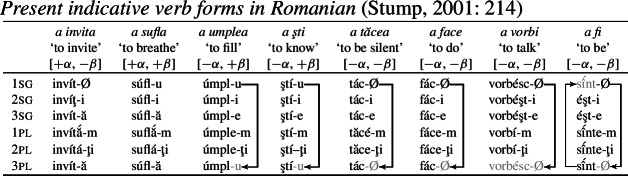

First, notice that there are two distinct forms for 1sg in the present indicative. For *a invita* (‘to invite’), for example, we see a zero ending and for *a sufla* (‘to breathe’), we find *-u*. In all verbs apart from these two, the 3rd plural is syncretic with whatever the 1st singular form is. Stump treats this as directional syncretism, that is, as spreading of the 1sg form to a 3pl context, illustrated by the arrows to the right of the forms in (82). Furthermore, with the verb *a fi* (‘to be’), we observe that the 1sg form is syncretic with the 3pl, which appears to unexpectedly take the plural stem. For this reason, Stump views this as a case of bidirectional syncretism (a divergent one in Baerman’s terms). We have spreading of the relevant 1sg marker to 3pl in six of the listed verbs (*a umplea*, *a şti*, *a tăcea*, *a face*, *a vorbi* and *a fi*), while what looks like the singular stem is also unexpectedly found in the 3pl with *a vorbi*.^18^ In addition, the stem form of *a fi* (‘to be’) appears to exhibit directional syncretism in the opposite direction (from 3pl to 1sg), thereby leading to bidirectionality in the paradigm.

This seems to pose a problem for the markedness restriction on bidirectional syncretism that we have been trying to maintain, as each take-over in the bidirectional pattern involves a feature change along two dimensions (person and number) rather than just one. Consequently, the spreading of *-u/-Ø* arguably involves an increase in markedness along the person dimension (3rd→1st), but a decrease along the number dimension (pl→sg). For the stem form of *a fi*, these relative markedness changes go in the opposite direction.

Recall that our solution to divergent BDS treats one directional syncretism as a genuine feature change to a less-marked feature combination, while the other is a retreat to the general case (i.e. impoverishment). The problem posed by the Romanian case is that, regardless of which directional syncretism we try to analyze as a markedness-reducing feature change, there will have to be an increase in markedness on at least one scale (either person or number).^19^ So, even for our approach, divergent BDS in Romanian appears to remain a problem.

Our response to this challenge, however, is to argue that it is not necessary to view the paradigm in (82) as bidirectional. Building on similar observations in Bobaljik (2002), we will show that this paradigm can be insightfully analyzed entirely in terms of impoverishment and underspecification alone.

First, Stump’s claim that the 1sg/3pl syncretism of *-u/-Ø* is directional is based on the assumption that these markers are fully specified for 1sg, as seems to be the case in *a invita* and *a sufla*. However, as already pointed out by Bobaljik (2002: 65), there are good reasons to want to treat *-u* as a default. For example, if we consider the imperfect paradigm in (83) (also discussed in Stump, 2001), we see that *-u* surfaces in the 3pl of *a cânta* (‘to sing’). The fact that it appears in 1sg and 3pl across the present indicative/imperfect paradigms can be taken to justify its status as a default exponent that is underspecified for person, number and tense.^20^

(83)
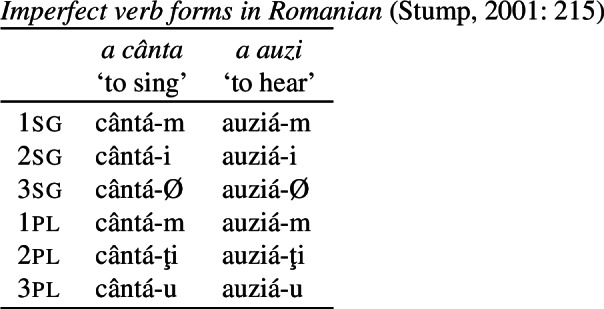

Furthermore, as Stump (2001: 215) mentions, *-m* is the 1sg form in the imperfect across all verb conjugations. While Stump asserts that *-m* should be treated as a 1pl (and thus would necessitate markedness-violating directional spreading to 1sg; Stump, 2001: 238), this is by no means a necessary assumption. Bobaljik (2002: 66) instead argues that *-m* can be treated as an exponent of 1st person that is underspecified for number, a distribution that we can clearly see in the imperfect in the paradigm (83).

Returning to the present indicative paradigm in (82), this means that the expected realization of *-m* in 1sg must be bled by the impoverishment rule in (84), which deletes the person and number values of 1sg in the present indicative (note that we do not attempt to further decompose person or tense features here for reasons of simplicity). This is an option that was also mentioned by Bobaljik (2002: 65).

(84)
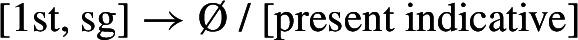

As a result, the expected 1st person marker in (85a) cannot be inserted in 1sg present indicative contexts (but will still surface in the imperfect). Instead, a less-specified class-specific exponent will be chosen. In (82), we see that the default is dependent on the particular verb: Some verbs take *-u*, while others take *-Ø* (again, an insight borrowed from Bobaljik, 2002). To account for this, we posit various inflectional classes of verbs in (82).^21^ Following previous work on transparadigmatic syncretism across inflection classes (Halle, 1992; Oltra Massuet, 1999; Müller, 2004a,b, 2005; Opitz, 2006; Alexiadou & Müller, 2008; also cf. Halle & Marantz, 2008), we assign all verbs in Romanian the abstract feature [±*β*], where [−*β*] characterizes the verbs with a *-Ø* default and [+*β*] singles out those verbs that take *-u* as their default form.

(85)

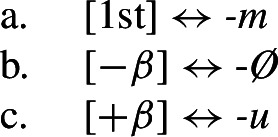

Now, we can turn to the apparent cases of directional syncretism in (82). Recall that we find 1sg/3pl syncretism in all verbs in (82) except for *a invita* and *a sufla* (traditional class 1). Since we have established that *-u/-Ø* are class-specific defaults, this putative directional syncretism is simply a retreat to the general case derived by impoverishment. In order to characterize the class of verbs participating in this syncretism, we use an additional decomposed inflection class feature [±*α*]. All verbs that exhibit this 1sg/3pl syncretism in the present indicative are associated with the feature [−*α*], which triggers the impoverishment rule in (86).

(86)
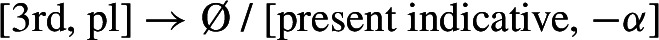

Given the entries in (85b,c), the relevant class-specific Elsewhere form (*-u/-Ø*) will surface in 3pl, too. Also, notice that there is a distinct 3sg present indicative form *-ă* (syncretic with 3pl) in [+*α*] contexts, further justifying the need for this feature.

Finally, let us consider *a vorbi* and *a fi*, both of which belong to the traditional fourth conjugation. In *a vorbi*, it is not just the suffix but also the stem that changes in 3pl, giving rise to whole-word syncretism (Stump, 1993). We can readily account for this if we assume that *vorbésc-* is the default stem (87b), while *vorbí-* is the dedicated plural stem (87a).

(87)
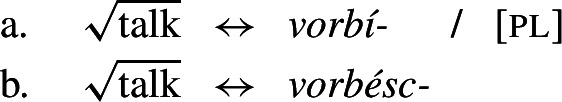

Thus, the insertion of the plural stem in (87a) will also be bled by the impoverishment rule in (86), in addition to the expected 3pl suffix.

Turning now to *a fi*, recall that the appearance of the stem form  in the 1sg was claimed to instantiate directional syncretism in the opposite direction (from 3pl→1sg), rendering this paradigm bidirectional. However, this stem change need not be directional syncretism at all. Given the fact that we have impoverishment across the 1sg in the present indicative, we can also treat the stem change as the emergence of a less-specific form. To account for the exceptionality of *a fi* (‘to be’), we can assume that this verb differs idiosyncratically from all others in having its plural stem as the default form, while other verbs have a specified plural stem and a singular Elsewhere, as in (87). Given the entries for the stem forms of *a fi* in (88), the insertion of the more specific singular stem in (88a) will be bled by the 1sg-impoverishment rule in (84) that deletes singular. The default plural stem in (88b) will be inserted instead.

(88)
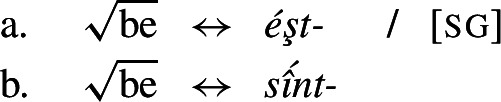

This accounts for the stem change in *a fi*, which was the sole motivating evidence for the bidirectionality of this pattern, as a product of a more general process of impoverishment applying to 1sg in the present indicative.

Consequently, we contend that the Romanian case, despite its apparent challenge for a markedness-decreasing condition on directional syncretism, can be argued not to involve bidirectional syncretism at all. In the following section, we will argue that this may well be the case for certain patterns of convergent BDS, too.

### Convergent bidirectional syncretism as unidirectional syncretism

Recall from Sect. 3.1 that a convergent bidirectional syncretism is observed when two instances of directional syncretism, i.e. take-overs, have different source categories but the same target category. One instance of this that we discussed before comes from declension in Bonan (20), repeated below as (89).

(89)
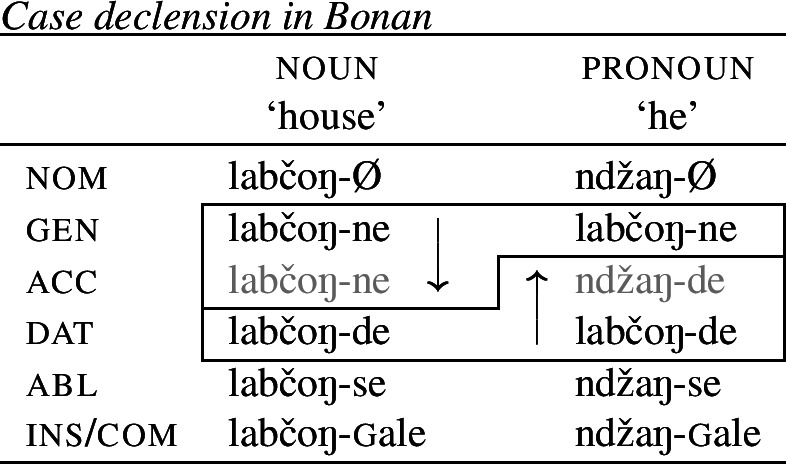

Recall that Harley (2008) captured this pattern by specifying *-ne* for a feature shared between the genitive and the accusative and *-de* for a feature shared between the dative and the accusative (90). As both markers are in principle compatible with an accusative context and are equally specific, this indeterminacy is resolved by each of the impoverishment rules in (91), applying in nouns and pronouns respectively.

(90)
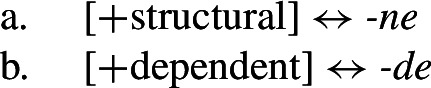

(91)
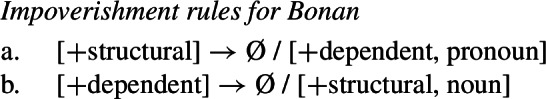

The result of these rules is shown again in (92).

(92)
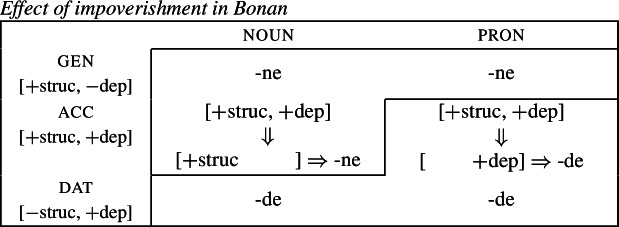

This analysis requires a separate impoverishment rule for each instance of directional spreading in addition to the assumption of a partially overlapping distribution for *-ne* and *-de*. Noyer’s approach to directional syncretism offers an alternative approach, however, which does not require these assumptions. Since it is possible to change a given feature specification into a contextually less-marked one, we can actually analyze the Bonan paradigm as unidirectional, thereby avoiding the need for overlapping marker distributions.

Let us assume that *-ne* is a fully specified genitive exponent (93a), while *-de* is underspecified as before (93b).

(93)
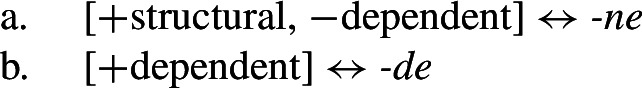

Provided that genitive is lower on the case hierarchy and thereby less marked than the accusative (cf. Starke, 2017, but see also Harðarson, 2016; Zompì, 2019; Bárány, 2021 for the notoriously difficult placement of the genitive on the hierarchy), we will assume that [+dependent] is the marked feature value in the context of [+structural] (94).

(94)
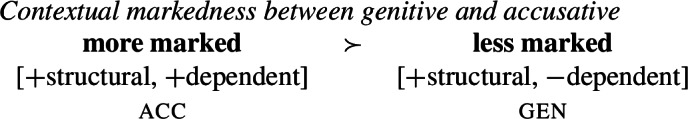

Now, we only need only one of the impoverishment rules in (91), namely (91b). This rule deletes the marked feature [+dependent] on nouns, which then triggers insertion of the unmarked feature [−dependent] by a persistent redundancy rule. This then effectively turns an underlying accusative context into a genitive one. Given the VIs in (93), the regular genitive marker *-ne* will be inserted into this accusative-turned-genitive context, as shown in (95). The insertion of *-de* in accusative pronouns is straightforward, as *-ne* does not fit this context.

(95)
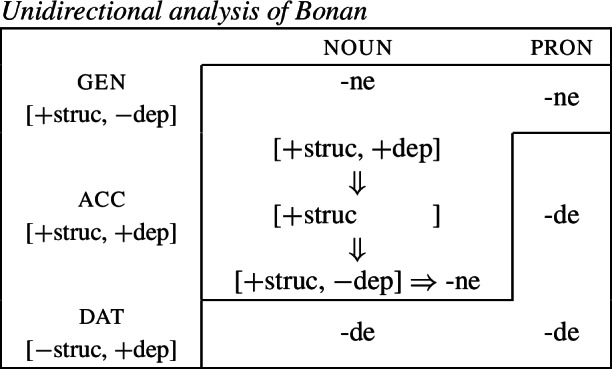

This analysis now treats the Bonan paradigm as involving unidirectional rather than bidirectional syncretism, as there is only one instance of directional spreading. This removes the indeterminacy of the previous analysis, as we can avoid adopting a (partially) overlapping specification for the two exponents. Instead, only one of the markers is underspecified, while the spreading form is fully specified, as in (94). Since feature-changing rules in Noyer’s system are restricted by markedness, it is only possible to change a particular feature specification into a less-marked one. Given the case hierarchy we are assuming here, this means that the actual directional syncretism must be from gen to acc. Treating *-de* as directional spreading would not be possible if dat is more marked than acc.

In principle, we could analyze all convergent BDS in a similar fashion, that is, as unidirectional syncretism. Furthermore, in any given case, we will know which form should be taken as the spreading one, as this will be given by the markedness relations between the relevant features. Since markedness-restricted feature insertion is already needed to account for divergent BDS, as we have argued for in Sect. 4.2, precisely the same analytical tools naturally lead to this unidirectional analysis of convergent BDS.

### Hidden bidirectional syncretism

In this section, we would like to suggest that bidirectional syncretism might in fact be more prevalent than is often assumed. In some cases, the familiar bidirectional pattern might be obscured by more specific exponents blocking the expected forms.

A potential example of this comes from the Kashmiri paradigm in (96). Here, the recent past forms for verbs of conjugation class III are syncretic with those of the indefinite past for conjugation class II for all persons, numbers, and genders. The indefinite past forms of conjugation class III are in turn identical to those of the remote past for conjugation II.

(96)

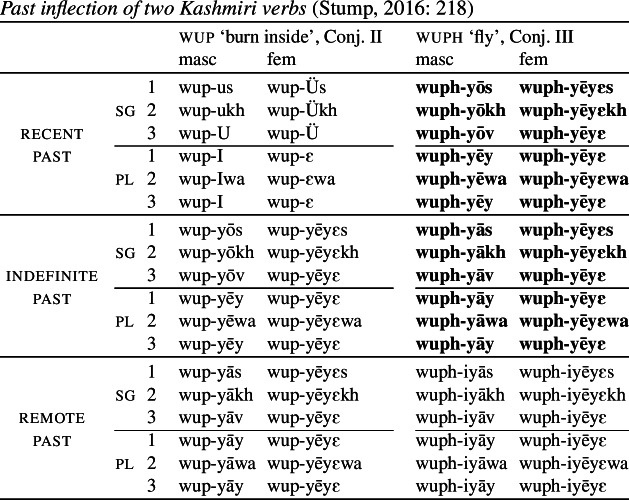

As summarized in (97), this paradigm appears to show diagonal syncretism, which is often indicative of an Elsewhere distribution (given that there can be no feature in common). The set of forms for indefinite past of conjungation II (B) are used for the recent past of conjungation III. Furthermore, the set of forms for the remote past of conjungation II (C) appear in the indefinite past of conjungation III.

(97)
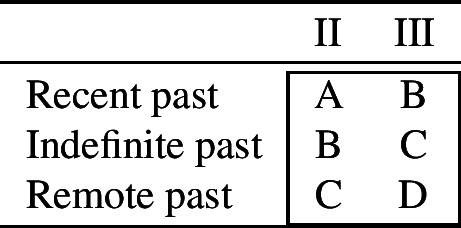

The B and C forms have diagonal distributions, meaning that both would have to be treated as Elsewhere forms (Baerman, 2004: 823–825). Given this problem, it has been argued that one of the two marker distributions requires reference to a non-syntactically motivated feature (see Stump, 2016: 217–223; cf. Baerman et al., 2005: 183–186 and Trommer, 2016: 74 on Dhaasanac). We would like to propose an alternative perspective in which neither the diagonal distribution of B or C is defined by a morphomic feature, but instead that this instantiates the familiar pattern of convergent bidirectional syncretism that is obscured on the surface by more specific forms (A and D) occupying the corner cells (98).

(98)

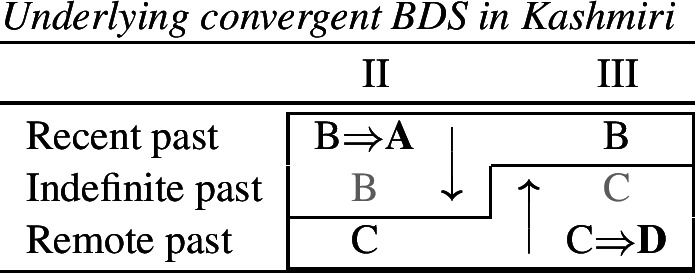

In analyzing Kashmiri, we will abstract away from the person, number and gender distinctions that make up the sub-paradigms that we have abbreviated as B and C and focus on providing specifications for these ‘blocks’. We encode the conjugation difference as an arbitrary class feature [±*α*] where conjugation II is [+*α*] and conjugation III is [−*α*]. For tense, we propose the following decomposition of the three past tenses in Kashmiri using the features [±recent] and [±remote]:

(99)
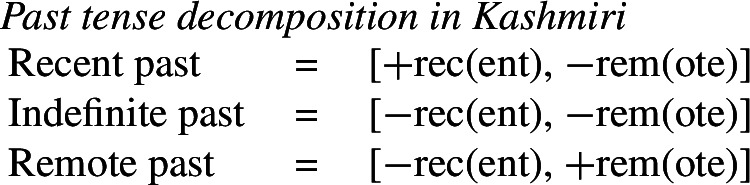

Recall from Sect. 4.5 that convergent BDS can be analyzed as unidirectional spreading of a fully specified marker in one of the contexts, with the other context is covered by an underspecified form. In Kashmiri, we treat the C forms as fully specified for remote past (100b). The B form is underspecified so it fits recent and indefinite contexts (100d). Both the A and D forms are fully specified for their respective cells (100a,b).

(100)
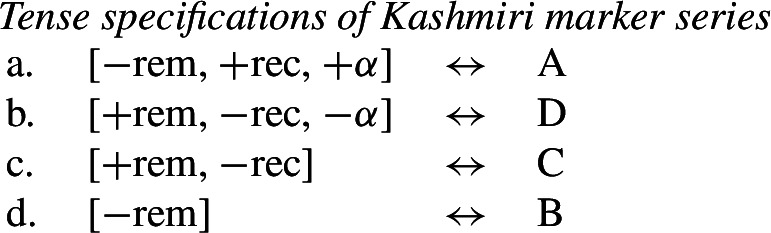

In order to derive the desired distribution of these exponents, we require the impoverishment rule in (101), which deletes [−rem] from indefinite past contexts in conjugation III.

(101)
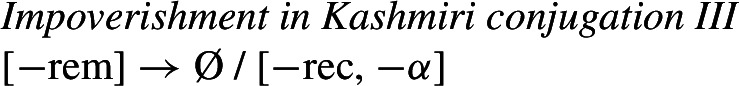

This rule leads to the spreading of C over the expected B in the indefinite past of conjungation III (−*α*), as shown in (102).

(102)

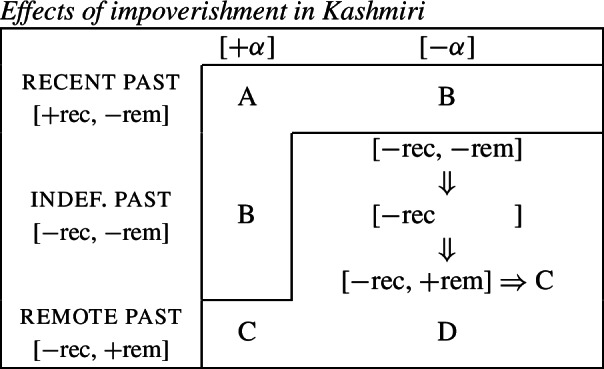

Assuming that a remote past is less marked than an indefinite past, this deletion triggers a redundancy rule in the indefinite past that inserts the feature [+rem], which is the unmarked feature in the context of [−rec]. This effectively turns an indefinite past into a remote past context. The central idea here is that C has spread from the remote past to the indefinite past in conj. III, even though it does not appear there on the surface (it is blocked by the more specific D form). The B form in the recent past of conj. II is blocked by A in a similar fashion.

This means that the apparently challenging diagonal distributions of B and C can actually be viewed as the familiar convergent BDS pattern, which is masked by more specific forms occupying the corner cells. This opens up the possibility of treating similar apparent cases of diagonal syncretism, which require recourse to morphomic features, as underlyingly bidirectional syncretisms. This is not to say, however, that all patterns that have been identified as morphomes can be treated in this way (see Maiden, 2018; Herce, 2023).

## Conclusion

In this paper, we have shown that, contrary to what has been claimed in previous literature, bidirectional syncretism can be readily accounted for in Distributed Morphology. While the divergent pattern had been correctly shown to resist an analysis in terms of underspecification and blocking alone (Baerman, 2004), once we adopt the refinements in Noyer (1998), where impoverishment may have two distinct outcomes, divergent BDS is no longer problematic. Crucially, these two differing results of impoverishment, a genuine markedness-reducing feature change on the one hand and a retreat to the general case on the other, are both found in divergent BDS. This analysis also allows us to maintain a version of the Unmarkedness Hypothesis, the idea that syncretism is universally markedness reducing, once it is stated over insertion contexts rather than exponents (*contra* Bobaljik, 2002). Despite the fact that divergent bidirectional syncretism clearly contradicts this hypothesis on a descriptive level, the analysis we propose involves a uniform decrease in markedness (a change from more marked to less marked and less marked to underdetermined).

The consequence of this is that it is not the case that the feature manipulation approach to syncretism in DM fails to capture these patterns. Furthermore, since Noyer’s approach is still less permissive than unrestricted rules of referral, one could argue, as some have, that the DM approach should be preferred on conceptual grounds (see e.g. Bobaljik, 2002). As a reviewer notes, however, the approach to syncretism developed here requires the additional postulation of markedness hierarchies and feature structures may not entirely come for free. Understandably, those working outside of DM may find that the reliance of the framework on such concepts somewhat undermines its apparent advantages vis-à-vis restrictiveness.

This also raises the question of how to test the restrictiveness of the DM approach to syncretism. There are well-documented patterns which are clear counterexamples, at least on a descriptive level, to the underlying assumptions about syncretism in DM (Baerman et al., 2005; Baerman, 2005), divergent bidirectional syncretism being one of them. As we have shown, these do not hold on the analytical level, however, meaning that any claims DM makes about restrictiveness will hold only of the implementation, not the surface description.

Does this then mean that DM does not make any clear empirical predictions? As we have seen, there are often several ways to analyze a given paradigm regarding what kind of syncretism is involved, e.g. uni- vs. bi-directional, directional vs. non-directional. What the DM approach does make clear, however, is what kind of analyses are not possible. A genuine falsification of the DM approach would involve a paradigm that could not be reconciled with the prevailing assumptions of the theory. For example, we have shown that divergent BDS must involve both a genuine feature change and a retreat to an Elsewhere case via impoverishment. If one could show that these assumptions could not be upheld, then this would be a genuine problem for DM. An abstract example of such a scenario is given in (103). If the familiar divergent BDS pattern were embedded in a larger paradigm containing another clear Elsewhere distribution (that of C), then it would seem to preclude the designation of either A or B as an Elsewhere form.

(103)
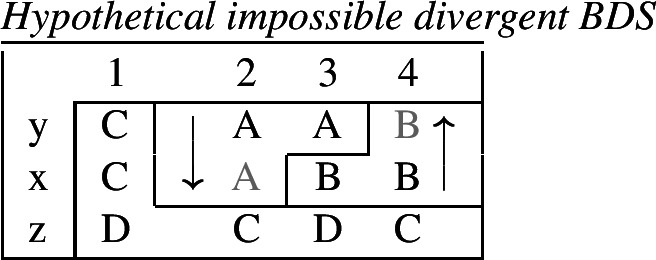

Here, there would appear to be little option other than to appeal to some morphomic feature for the distribution of C, a move that would clearly be undesirable in an analysis which seeks to make use of only syntacticosemantic features. For a rule of referral approach, on the other hand, the paradigm in (103) would be entirely unproblematic.

Overall, we hope that this paper highlights the importance of discourse between those working in different theoretical frameworks. It is clearly not a good reflection on the field that the challenge of bidirectional syncretism was not responded to, at least directly, by those working in DM. Understandably, this can lead to the impression among those skeptical of DM that its practitioners simply ignore the valid objections raised by those working outside the framework (Spencer, 2019: 44). For what it is worth, we believe there might be another reason for this disconnect, namely that the syntacticocentric nature of the theory has often led researchers to different kinds of questions, in particular to those relating to the mapping of syntactic structures to word forms, i.e. the nature of postsyntactic operations (e.g. Embick & Noyer, 2001; Arregi & Nevins, 2012) or locality conditions on contextual allomorphy (e.g. Bobaljik, 2000, 2012; Embick, 2010). In a theory that treats paradigms as epiphenomenal, it is perhaps not all that surprising that detailed investigations of paradigm structure have been somewhat neglected.

There has been sporadic cross-framework dialogue that has undoubtedly led to fruitful exchange, see e.g. Bobaljik (2002) and Stump (2001), Harley (2008) and Baerman et al. (2002), Spencer (2000) and Bobaljik and Branigan (2006), as well as Müller (2007) and Halle and Marantz (2008) responding to Carstairs-McCarthy (1994) and Cameron-Faulkner and Carstairs-McCarthy (2000), respectively. We therefore agree with Kramer (2016: 117) that the field would stand to benefit from more frequent ‘stepping across the aisle’ to pursue the shared goal of a better understanding of morphological systems.

## Footnotes

1 Bidirectional syncretism has been mentioned sporadically in the DM literature as a potential problem (see e.g. Albright & Fuß, 2012; Kramer, 2016), but has not yet received a detailed treatment. Müller (2013) takes the challenging nature of BDS as justification to depart from certain core assumptions of DM. As we will discuss in Sect. 3.1, a case of convergent BDS has actually already been analyzed in Harley (2008), but the connection to bidirectional syncretism was not made there.

2 For reasons of space, we do not include the relevant feature values for gender, e.g. [±masc, ±fem], or animacy ([±human]) in the cell specifications.

3 Throughout the paper, we do not include the typical underscore () in the contextual specification of such rules. This is sometimes useful in order to indicate whether the conditioning features are located in the same feature bundle ([a, b, ]) or on an external head ( [a, b]). Since this will not be relevant in the present discussion, we decided to omit this detail.

4 Stump is not explicit about which marker would be expected here, but we can assume, as we have in (12a), that *-š* is a general 2nd singular marker, similar in distribution to 2nd plural *-te*. Note that the VI in (12b) constitutes inwardly-sensitive grammatically conditioned allomorphy. This is incompatible with the argument presented in Bobaljik (2000) that inward sensitivity of allomorphy for grammatical features is impossible due to the rewriting of features by phonological forms. While this might appear to cast doubt on the analysis involving (12b), it is worth mentioning that Bobaljik’s (2000) argument has been challenged in subsequent work (e.g. Carstairs-McCarthy, 2001; Gribanova & Harizanov, 2017; Winchester, 2017).

5 Of course, this problem only exists if the solution must be morphological. As a reviewer notes, there have been other instances of apparently difficult syncretisms that have received alternative, non-morphological, explanations, see e.g. Kalin (2015) on morphological reversals (cf. Baerman, 2007), Embick (1997) and Lee (2015) on voice syncretism, and Lecarme (2002) and Kramer (2015) on Somali plurals. For the cases of bidirectional syncretism that we will discuss, however, we cannot see a plausible alternative syntactic/semantic account of the facts, so will continue to assume that the solution must be morphological in nature.

6 An apparently similar situation has been described for so-called ‘ambigeneric’ (neuter) nouns in Romanian, which appear to take the masculine ending with singular nouns and the feminine ending with plurals (see Kramer, 2015; Matushansky, 2022; Kramer & Sande, 2023). This pattern could therefore also be viewed as convergent bidirectional syncretism, albeit certain cells blocked by more specific exponents (see the discussion of Kashmiri in Sect. 4.6). However, note that Kramer (2015) actually analyzes this pattern as spreading of the neuter forms in singular and plural, i.e. as a non-convergent bidirectional syncretism. To uphold a convergent BDS analysis of this pattern, one would have to adopt a different feature decomposition of gender, in particular we would require two binary gender features (e.g. [±fem, ±masc]) and neuter could no longer be treated as the absence of gender.

7 Harley encodes the distinction between nouns and (local) pronouns via a [+participant] feature. The exact impoverishment rule therefore deletes [+structural] in the context of [+dependent, +participant].

8 It should be noted, however, that what is described as divergent BDS as per the definition in (34) is more specific than this. To qualify as divergent BDS, it must be the case that the target of one directional syncretism in the paradigm must be the source of the other, and *vice versa*. As such, this definition of divergent BDS seems to imply some kind of polarity. In principle, a pattern of bidirectional syncretism could still be ‘divergent’ in the sense that it is non-convergent (each directional syncretism has a distinct target).

9 Notice that this decomposition of case differs slightly from what Harley assumed for Bonan. There, nominative and accusative are both [+structural], but are distinguished by the feature [±dependent] rather than [±superior]. For present purposes, this derives the same result if we replace [+superior] with [−dependent] in the nominative, for example. To capture the full Latin paradigm, Halle also assumes the feature [±oblique] which is not directly relevant here.

10 An alternative technical solution would be to treat one of the exponents, e.g. *-us*, as an underspecified Elsewhere exponent for case. This would mean that *-um*, specified for [+structural], would be expected to appear in all nom/acc contexts. Thus, each occurrence of *-us* (including in class II) would have to be effected by an impoverishment rule. As a reviewer notes, this is essentially an analysis in which the morphology treats nominative as a default form (appearing only as the result of impoverishment). The consequence of this analysis is that there would be no nominative forms as such in the morphological system. In the more elaborate divergent BDS in Nimboran that we will discuss in Sect. 4.1, this would mean that the apparent dual marker *-k*, for example, is also treated as an Elsewhere (with *-i* realizing non-singular). A potential problem with this approach, as far as we can tell, comes from learnability. If, as Pertsova (2007) has claimed, a learner will first posit fully specified forms, when they encounter an exponent such as *-us* in nominative contexts for all masculine nouns (class II) and in nominative and accusative for a smaller group of neuter nouns (class III), then there would be little motivation to assume that *-us* is a radically underspecified default case form rather than simply underspecified for the nom/acc distinction.

11 Interestingly, while an underspecification plus blocking/impoverishment approach can capture convergent but not divergent BDS, the more restricted directional approach proposed by Stump (2001), namely the *Bidirectional Referral Principle*, has the opposite empirical coverage. For purely technical reasons, it can derive divergent BDS, yet struggles with the convergent pattern (see Müller, 2008: 210–213 for detailed discussion).

12 Note that we have simplified the paradigm here somewhat by omitting the additional prefix *maN-* that appears in 1.incl.sg contexts. Furthermore, the 1st singular inclusive has a dual interpretation, even though it lacks the dual suffix -*k*.

13 We could equally use [±speaker] and [±hearer] as we do later in the paper for languages with a clusivity distinction in person.

14 In non-impoverished plural contexts, i.e. [−sg, +pl], both of the markers in (58) are equally good candidates for insertion given the Subset Principle (Halle & Marantz, 1993) as each realizes one of the sub-features of the context. In order for ^*-i*^ to block *-k* in general, we can appeal to extrinsic ordering or a hierarchy of sub-features [±pl] ≻ [±sg].

15 Noyer also discusses the fact that the A- and C-stems look as if they are phonologically derived from the B-stem (which justifies its position as the elsewhere candidate) by metathesis and ablaut respectively. We could also assume that the feature specifications in (63) trigger these special phonological rules, but refrain from doing so here (Inkelas, 1993; Keine, 2013).

16 It is clear that something else must be said about why gender distinctions are not present in local persons (1st/2nd). If gender features are not restricted to 3rd person in the lexicon or the syntax, then complete neutralization of gender distinctions for local persons ([±masc] → Ø/ [+1/+2]), too (see Harley, 2008: 277 for a similar approach to the lack of gender distinctions in non-third person English pronouns).

17 For example, Stump’s (2001) formalization of rules of referral in the first version of Paradigm Function Morphology treats directional rules as a kind of counterfactual feature change. Whatever rule would normally apply for a given cell is blocked by a rule of referral that mirrors the output of whatever rule would have applied if the cell’s feature specification had differed with regard to some particular feature(s). For divergent BDS in Latin, the spreading from nominative to the accusative in class III could be achieved by the rule of referral in (82) that essentially treats the class III accusative cell ([−sup, +struc]) as if it had [+sup] instead.

As far as we can tell, this implementation does not mimic the effects of deletion of features, however. This is what would be required for the other directional syncretism in class I (nom→acc). The basic problem here, as mentioned above, is that rules of referral manipulate ‘addresses’ (i.e. paradigm cells) rather than feature sets.

18 This might also be the case for the other verbs too, but it is possible that the difference in the 1pl and 2pl stems is the result of phonologically-conditioned stem allomorphy due to the consonant-initial endings attaching to a consonant-final stem (compare *şti-* where we see no difference).

19 One might try to manipulate the dimensions independently such that there is a feature change by impoverishment and subsequent redundancy-driven insertion for one dimension (i.e. a step from *most marked* to *less marked*), while there is plain impoverishment in the other (i.e. a step from *less marked* to *least marked*), cf. (81). Take for example the spreading of *-u/-Ø* which requires a 3→1 change in person and a pl→sg change in number and thus the two impoverishment rules in (83), the second of which will be immediately followed by a persistent redundancy rule inserting [−pl].

As is evident, regardless of which impoverishment rule applies first, it necessarily bleeds the subsequent one (an instance of mutual bleeding). Note that dropping the problematic [−speaker] feature from the contextual specification of (82b) is not an option. This would expand (82b)’s application domain to also include 1pl giving rise to an unobserved syncretism between 1sg and 1pl. The same problem holds, *mutatis mutandis*, for other orders of application and the other directional syncretism.

20 As a reviewer notes, an alternative interpretation would be that these are unrelated forms (accidental homophony). However, we adhere to Müller’s (2005) *Syncretism Principle*, which states that ‘identity of form implies identity of function’. Accordingly, one should treat even transparadigmatic syncretism as non-accidental.

21 Note that our decomposition does not fully correspond to the four traditional conjugation classes in Romanian that Stump adopts. These classes are based on the infinitive forms of verbs rather than the distribution of the markers in the finite paradigms and are therefore not able to capture the syncretisms in (82). The traditional conjugation class 1 corresponds to our [+*α*] specification, while class 4 is uniquely identified by [−*α*,−*β*]. Class 2 (*a umplea* and *a tăcea*) and class 3 (*a face*, *a şti*) do not form natural classes on our decomposition (precisely because the distribution of *-u/-Ø* cross-cuts this distinction). If we still wanted to define these classes, e.g. in order to determine the inflection of the infinitival forms, then we would require a further decomposition of inflection class to include an additional feature such as [±*γ*].
